# LINC00473‐modified bone marrow mesenchymal stem cells incorporated thermosensitive PLGA hydrogel transplantation for steroid‐induced osteonecrosis of femoral head: A detailed mechanistic study and validity evaluation

**DOI:** 10.1002/btm2.10275

**Published:** 2021-12-08

**Authors:** Yingxing Xu, Yaping Jiang, Yingzhen Wang, Bin Jia, Song Gao, Haiyang Yu, Haining Zhang, Chengyu Lv, Haiyan Li, Tao Li

**Affiliations:** ^1^ Department of Joint Surgery The Affiliated Hospital of Qingdao University Qingdao China; ^2^ Department of Medicine Qingdao University Qingdao China; ^3^ Department of Oral Implantology The Affiliated Hospital of Qingdao University Qingdao China; ^4^ Department of Radiology The Affiliated Hospital of Qingdao University Qingdao China

**Keywords:** bone marrow mesenchymal stem cells, cell therapy, hydrogels, LncRNA LINC00473, osteonecrosis of femoral head

## Abstract

The pathogenesis of steroid‐induced osteonecrosis of the femoral head (SONFH) involves a glucocorticoid‐induced imbalance of osteogenic and adipogenic differentiation, and apoptosis of bone marrow mesenchymal stem cells (BMSCs). An increasing number of genes, especially noncoding RNAs, have been implicated in the function of BMSCs. Our previous studies have confirmed the key role of LINC00473 and miR‐23a‐3p on the osteogenic, adipogenic differentiation, and apoptosis of BMSCs. However, the underlying mechanism of this process is still unclear. Based on bioinformatics analysis, here we investigated the effects of LINC00473 on the LRP5/Wnt/β‐catenin signaling pathway in the osteogenesis and adipogenesis of BMSCs, as well as the PEBP1/Akt/Bad/Bcl‐2 signaling pathway in dexamethasone‐ (Dex‐) induced apoptosis of BMSCs. Our data showed that LINC00473 could promote osteogenesis and suppress the adipogenesis of BMSCs through the activation of the miR‐23a‐3p/LRP5/Wnt/β‐catenin signaling pathway axis, while rescuing BMSCs from Dex‐induced apoptosis by activating the miR‐23a‐3p/PEBP1/Akt/Bad/Bcl‐2 signaling pathway axis. Notably, we observed that LINC00473 interacted with miR‐23a‐3p in an Argonaute 2 (AGO2)‐dependent manner based on dual‐luciferase reporter assay, AGO2‐related RNA immunoprecipitation, and RNA antisense purification assay. Furthermore, injectable thermosensitive polylactic‐co‐glycolic acid (PLGA) hydrogel loaded with rat‐derived BMSCs (rBMSCs) modified by LINC00473 were used for the treatment of SONFH in a rat model. Our results demonstrated that PLGA hydrogels provided a suitable environment for harboring rBMSCs. Besides, transplantation of PLGA hydrogels loaded with rBMSCs modified by LINC00473 could significantly promote the bone repair and reconstruction of the necrotic area at the femoral head in our SONFH rat model. Surprisingly, compared with the transplantation of BMSCs alone, the transplanted rBMSCs encapsulated within the PLGA hydrogel could migrate from the medullary cavity to the femoral head. In summary, LINC00473 promoted osteogenesis, inhibited adipogenesis, and antagonized Dex‐induced apoptosis of BMSCs. Therefore, LINC00473 could provide a new strategy for the treatment of SONFH.

## 1 INTRODUCTION

Steroid‐induced osteonecrosis of the femoral head (SONFH) is a progressive disease characterized by necrosis of the subchondral bone, accompanied by severe hip pain and dysfunction.^1^ As the dominant treatment for SONFH, total hip arthroplasty (THA) can significantly relieve pain and improve the hip function for patients with SONFH. However, the results of THA in young patients are often hindered due to prosthesis‐related complications.^2^, ^3^ In addition, hip‐preserving treatment for SONFH also is not satisfactory.^4^, ^5^, ^6^, ^7^, ^8^, ^9^

The limited treatment for SONFH is attributed to unclear pathogenesis of this disease. It is thought that vascular compromise, cell apoptosis, deficient bone repair, oxidative stress, and disturbances of lipid metabolism are key elements in the development of SONFH.^10^ Among these, the glucocorticoid‐induced imbalance of osteogenic and adipogenic differentiation, and apoptosis of bone marrow mesenchymal stem cells (BMSCs) have brought widespread interest. On the one hand, glucocorticoid can induce BMSCs to differentiate into osteoblasts and chondrocytes^11^, ^12^ but this effect is concentration‐ and time‐dependent. Long‐term exposure to high doses of glucocorticoid can disturb the balance of osteogenic and adipogenic differentiation of BMSCs, inhibit cell proliferation, and induce the apoptosis of BMSCs,^13^, ^14^ which may be relevant to the pathogenesis of SONFH.

Various signaling pathways are involved in the biological function of BMSCs, such as the Wnt/β‐catenin,^15^ TGF‐β/BMPs,^16^, ^17^ and MAPK^18^ signaling pathways. However, many upstream genes selectively regulate these pathways that have not been explored with regards to SONFH. Among these upstream genes, noncoding RNAs (ncRNAs) have been proposed to be critical regulators of downstream gene expression, although they do not code proteins.^19^ Long noncoding RNAs (lncRNAs) are ncRNAs with more than 200 nucleotides and are capable of affecting the differentiation, proliferation, and apoptosis of BMSCs.^20^ It was reported that osteogenic and adipogenic differentiation of BMSCs could be regulated by several lncRNAs, including lncRNA RP11‐154D6,^21^ lncRNA TCONS_00041960,^22^ and LncRNA LOXL1‐AS1.^23^ Other lncRNAs, such as lncRNA‐p21,^24^ lncRNA HULC,^25^ and lncRNA NORAD^26^ have been confirmed to regulate the proliferation and apoptosis of BMSCs.

MicroRNAs (miRNAs), a type of small‐molecule noncoding RNA with 17‐25 nucleotides, play a key regulatory role in various cellular processes through their binding to the complementary 3′‐untranslated region of targeted mRNAs and thereby inhibition of post‐transcriptional expression.^27^, ^28^ The regulatory effects of miRNAs on BMSCs depend on their target genes. miR‐20b was found to inhibit adipogenic differentiation of BMSCs by binding to its target gene PPAR‐γ, and promoting the osteogenic differentiation of BMSCs through the transcription of Runx2.^29^ In addition, it was reported that miR‐29a could inhibit the expression of DKK1 and activate the Wnt/β‐catenin signaling pathway, and thereby promote the osteogenesis of BMSCs.^30^ On the contrary, several miRNAs, such as miR‐138, miR‐139, and miR‐133, inhibit the osteogenic differentiation of BMSCs.^31^, ^32^, ^33^

Notably, lncRNAs and miRNAs can interact to regulate cellular processes. For instance, lncRNAs can act as competing endogenous RNAs (ceRNAs) to bind to specific miRNAs and regulate their functions.^34^, ^35^, ^36^ Despite all this, our current knowledge of the roles of lncRNAs in SONFH remains limited.

According to our previous microarray analysis, miR‐23a‐3p was identified as an up‐regulated differentially expressed miRNA in dexamethasone‐induced BMSCs,^37^ and was confirmed to target low‐density lipoprotein receptor‐related protein 5 (LRP5) to inhibit osteogenic differentiation of BMSCs through the inactivation of the Wnt/β‐catenin signaling pathway.^38^ In addition, it has been reported that the inhibition of miR‐23a‐3p could decrease osteonecrosis incidence in a rat model.^39^ We also identified differentially expressed lncRNAs in BMSCs from patients with SONFH, and found that LINC00473 was significantly down‐regulated and could rescue BMSCs from dexamethasone‐induced apoptosis through the activation of the PEBP1/Akt/Bad/Bcl‐2 signaling pathway.^40^, ^41^ Furthermore, bioinformatics analysis based on the Encyclopedia of RNA Interactomes (ENCORI; http://starbase.sysu.edu.cn/index.php)^42^ showed that both miR‐23a‐3p, LRP5 and PEBP1 are involved in the ceRNA interaction network of LINC00473. Therefore, there may be a cascade reaction between LINC00473 and both miR‐23a‐3p/LRP5/Wnt/β‐catenin and miR‐23a‐3p/PEBP1/Akt/Bad/Bcl‐2 signaling pathway axes. In view of this, this study was designed to investigate the above predictions. Our data showed that LINC00473 could bind miR‐23a‐3p in an Argonaute 2 (AGO2)‐dependent manner. It also promoted osteogenic differentiation and suppressed the adipogenic differentiation of BMSCs through the activation of LRP5/Wnt/β‐catenin signaling pathway. LINC00473 was also shown to rescue BMSCs from the Dex‐induced apoptosis through the activation of PEBP1/Akt/Bad/Bcl‐2 signaling pathway.

Moreover, our previous study has confirmed that LINC00473 overexpression has no effect on the proliferation and cell cycle of human‐derived bone marrow mesenchymal stem cells (hBMSCs),^39^ which was positive for maintaining the stemness of BMSCs and achieving the best therapeutic effect in the cell therapy. Accordingly, the role of LINC00473 in stem cell therapy for SONFH was further explored in the present study. To our knowledge, stem cell transplantation combined with tissue engineering has attracted increasing interest and shown a promising prospect in the treatment of SONFH.^43^, ^44^ Injectable thermosensitive PLGA hydrogels were often used as cell‐delivery carrier in the tissue engineering,^45^, ^46^ due to the excellent biocompatibility, convenience, and minimally invasive procedure. However, the fate of transplanted stem cells in vivo is still controversial. It is uncertain whether the transplanted cells will spread with blood flow after transplantation, and undergo mutation, apoptosis, or even necrosis. Therefore, we further assessed the effects of the transplantation of PLGA hydrogels loaded with BMSCs modified by LINC00473 on SONFH in a rat model as well as the fate of the transplanted cells. We observed that this method could significantly attenuate the progression of SONFH in vivo. Surprisingly, transplanted rat‐derived BMSCs (rBMSCs) encapsulated within the PLGA hydrogel could migrate from the medullary cavity to the femoral head. In summary, our data propose novel aspects for the pathogenesis of SONFH, and provide a reference for the exploration of hip‐preserving surgery to treat SONFH.

## 2 RESULTS

### 2.1 The ceRNA interaction network of LINC00473

**FIGURE 1:**
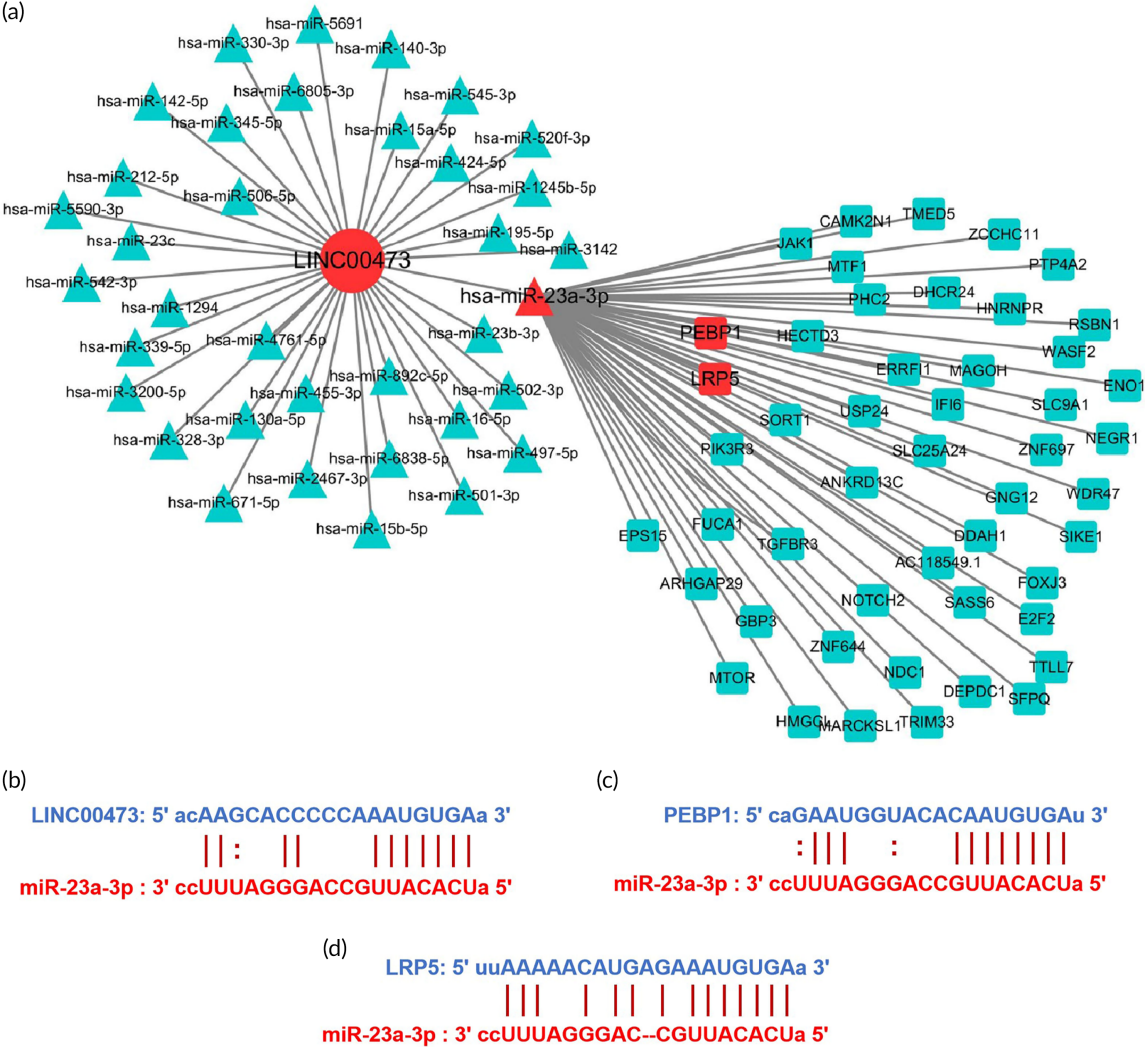
The ceRNA interation network of LINC00473. (a) The network diagram of LINC00473 as a ceRNA (only 50 target mRNAs for miR‐23a‐3p were selectively included due to the limitation of space); (b) The predicted binding sites of miR‐23a‐3p on LINC00473; (c) The predicted binding sites of PEBP1 on miR‐23a‐3p; (d) The predicted binding sites of LRP5 on miR‐23a‐3p. All data were obtained from the ENCORI database. ceRNA, competing endogenous RNA; ENCORI, encyclopedia of RNA interactomes; LINC00473, LINCRNA‐00473; miR‐23a‐3p, MicroRNA‐23a‐3p; LRP5, low‐density lipoprotein receptor‐related protein 5; PEBP1, phosphatidylethanolamine‐binding protein 1

To explore the regulatory mechanisms of LINC00473 in BMSCs, we constructed the ceRNA interaction network of LINC00473 by predicting its target mRNAs and miRNAs based on data from ENCORI (Figure 1a). A total of 36 miRNA‐targeting sites on LINC00473 were identified and, among them, miR‐23a‐3p was reported to inhibit osteogenic differentiation of BMSCs in our previous study^38^ (the predicted binding sites of miR‐23a‐3p on LINC00473 are shown in Figure 1b, and the correlation between them in 32 types of cancer are shown in Table 1). Furthermore, we detected a total of 3681 mRNA‐targeting sites on miR‐23a‐3p, including LRP5 and PEBP1. We have shown previously that LRP5 is a target of miR‐23a‐3p and that it inhibits osteogenic differentiation of BMSCs^38^ (the predicted binding sites of LRP5 on miR‐23a‐3p are shown in Figure 1c). Moreover, we have shown that PEBP1 is involved in the antagonism of LINC00473 on Dex‐induced apoptosis of BMSCs^41^ (the predicted binding sites of PEBP1 on miR‐23a‐3p are shown in Figure 1d). In addition, we also detected mRNA‐targeting sites on LINC00473, including only ZNF639 and C8orf34, not LRP5 and PEBP1. In view of this, there may be a cascade reaction between LINC00473 and both the miR‐23a‐3p/LRP5/Wnt/β‐catenin and miR‐23a‐3p/PEBP1/Akt/Bad/Bcl‐2 signaling pathways.

**TABLE 1:** Correlation between LINC00473 and miR‐23a‐3p in 32 types of cancer

Cancer	Cancer full name	Sample number	Coefficient‐*R*	*P*‐value	FDR
MESO	Mesothelioma	86	−0.472	4.38e−6	5.79e−4
CHOL	Cholangiocarcinoma	36	−0.362	2.99e−2	7.48e−1
ESCA	Esophageal carcinoma	162	−0.280	3.10e−4	9.98e−4
STAD	Stomach adenocarcinoma	372	−0.259	4.05e−7	8.90e−6
DLBC	Lymphoid neoplasm diffuse large B‐cell lymphoma	47	−0.255	8.32e−2	3.43e−1
PAAD	Pancreatic adenocarcinoma	178	−0.251	7.33e−4	2.85e−3
THCA	Thyroid carcinoma	509	−0.232	1.23e−7	1.25e−6
SARC	Sarcoma	261	−0.149	1.59e−2	3.32e−2
THYM	Thymoma	119	−0.142	1.24e−1	1.88e−1
LUSC	Lung squamous cell carcinoma	475	−0.128	5.11e−3	9.91e−3
UCEC	Uterine corpus endometrial carcinoma	538	−0.122	4.51e−3	5.41e−2
KIRP	Kidney renal papillary cell carcinoma	289	−0.099	9.15e−2	3.66e−1
READ	Rectum adenocarcinoma	161	−0.057	4.72e−1	5.37e−1
BLCA	Bladder urothelial carcinoma	408	−0.055	2.64e−1	3.83e−1
ACC	Adrenocortical carcinoma	79	−0.054	6.37e−1	9.59e−1
LUAD	Lung adenocarcinoma	512	−0.015	7.34e−1	8.28e−1
PCPG	Pheochromocytoma and paraganglioma	183	−0.013	8.61e−1	9.63e−1
HNSC	Head and neck squamous cell carcinoma	497	−0.004	9.34e−1	9.70e−1
CESC	Cervical squamous cell carcinoma and endocervical adenocarcinoma	306	0.002	9.66e−1	9.81e−1
COAD	Colon adenocarcinoma	450	0.007	8.81e−1	9.31e−1
KIRC	Kidney renal clear cell carcinoma	517	0.008	8.49e−1	9.01e−1
OV	Ovarian serous cystadenocarcinoma	376	0.011	8.30e−1	8.98e−1
UCS	Uterine carcinosarcoma	56	0.016	9.10e−1	9.67e−1
SKCM	Skin cutaneous melanoma	449	0.030	5.28e−1	7.26e−1
LGG	Brain lower grade glioma	525	0.055	2.04e−1	2.45e−1
PRAD	Prostate adenocarcinoma	495	0.072	1.12e−1	2.03e−1
LAML	Acute myeloid leukemia	83	0.083	4.56e−1	7.26e−1
BRCA	Breast invasive carcinoma	1085	0.102	7.88e−4	1.82e−3
UVM	Uveal melanoma	80	0.146	1.96e−1	4.64e−1
KICH	Kidney chromophobe	65	0.195	1.20e−1	3.02e−1
LIHC	Liver hepatocellular carcinoma	370	0.225	1.19e−5	5.88e−5
TGCT	Testicular germ cell tumors	156	0.242	2.38e−3	8.25e−3

### 2.2 Acquisition and identification of hBMSCs

**FIGURE 2:**
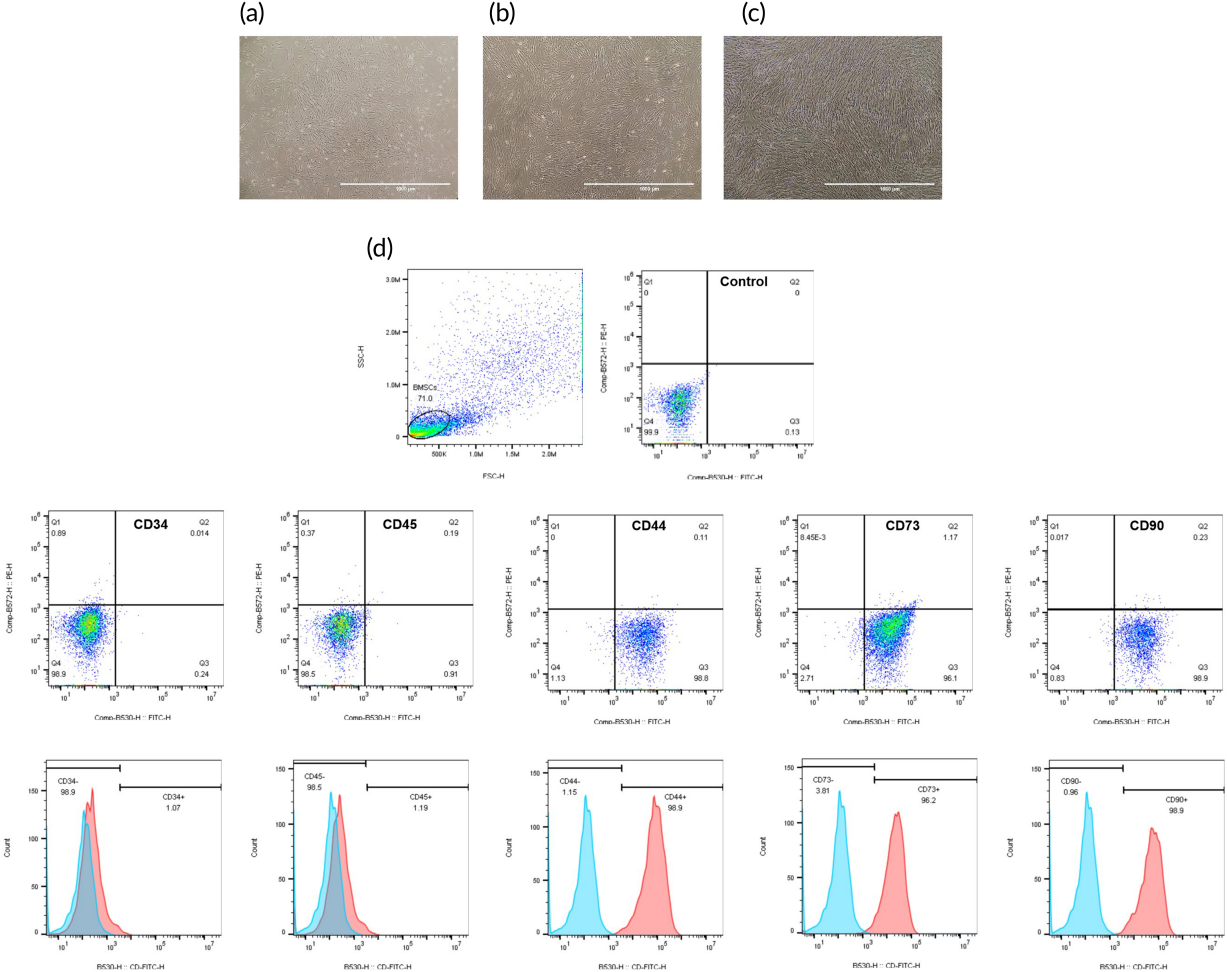
Identification of hBMSCs. (a–c) Representative images showing the morphology of hBMSCs under an inverted phase contrast microscope (scale bar = 1000 μm): (a) the first passage cells reached 80% confluence; (b) the second passage cells reached 90% confluence; (c) the third passage cells reached 100% confluence. (d) The surface markers of hBMSCs by flow cytometry analysis: CD34, CD44, CD45, CD73 and CD90. hBMSCs, human‐derived bone marrow mesenchymal stem cells

The hBMSCs were isolated from the bone marrow tissue, and were purified and expanded in vitro, according to a previous description.^40^ After three passages, the cells showed a fibroblast‐like phenotype, whirlpool‐like growth, and spindle‐shaped morphology (Figure 2a–c). Furthermore, the phenotyping of BMSCs suggested that the isolated cells expressed two typical surface markers for mesenchymal cells, namely, CD44 (98.8%), CD73 (96.1%), and CD90 (98.9%), and did not express two typical surface markers for hematopoietic cells, namely, CD34 (98.9%) and CD45 (98.5%) (Figure 2d). Therefore, the third passage cells were used in the following experiments.

### 2.3 LINC00473 as a master regulator of osteogenic differentiation and adipogenic differentiation of hBMSCs

**FIGURE 3:**
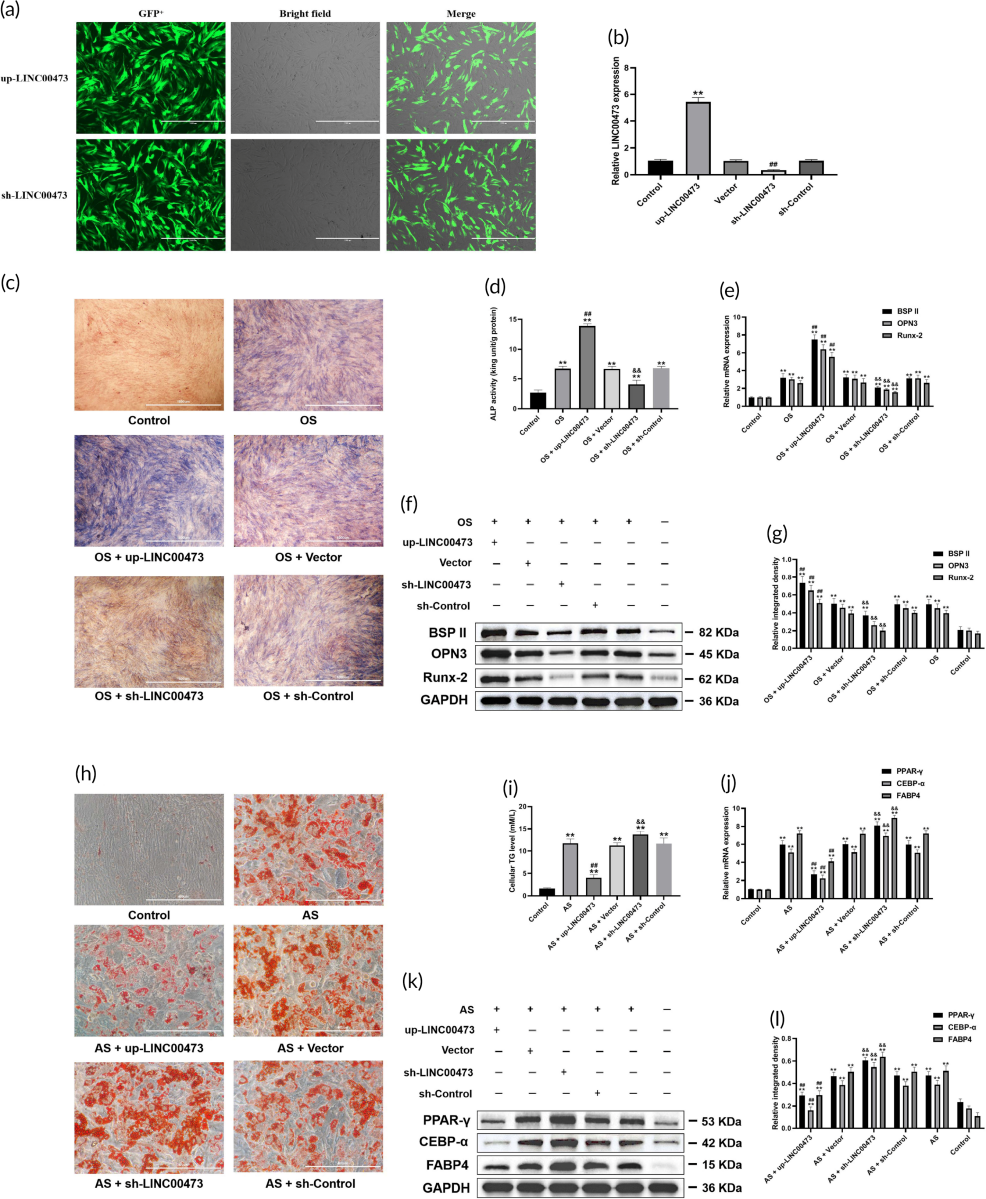
The effects of LINC00473 on the osteogenic and adipogenic differentiation of hBMSCs. (a) Efficiency of transfection in hBMSCs for LINC00473 under a fluorescence microscope (scale bar = 1000 μm). (b) Efficiency of transfection in hBMSCs for LINC00473 determined by qRT‐PCR analysis. (c) ALP staining for osteogenic differentiation of hBMSCs under an inverted phase contrast microscope (scale bar = 1000 μm). (d) ALP activity assay for osteogenic differentiation of hBMSCs. (e) The result of qRT‐PCR showing the mRNA expression levels of BSPII, OPN3, and Runx‐2 in each group. (f) The result of western blotting showing the protein expression levels of BSPII, OPN3, and Runx‐2 in each group. (g) The quantification of integrated density in target bands normalized to GAPDH. (h) Oil red O staining for adipogenic differentiation of hBMSCs under an inverted phase contrast microscope (scale bar = 400 μm). (i) Cellular TG content assay for adipogenic differentiation of hBMSCs. (j) qRT‐PCR showing the mRNA expression levels of PPAR‐γ, CEBP‐α, and FABP4 in each group. (k) Western blotting showing the protein expression levels of PPAR‐γ, CEBP‐α, and FABP4 in each group. (l) The quantification of integrated density in target bands normalized to GAPDH. Data are presented as mean value ± standard deviation of three independent experiments. (b) ^**^ *P* < 0.01 compared with the Control group and Vector group, and ^##^ *P* < 0.01 compared with the Control group and sh‐Control group. (d,e,g) ^**^ *P* < 0.01 compared with the Control group, ^##^ *P* < 0.01 compared with the OS group and OS + Vector group, and ^&&^ *P* < 0.01 compared with the OS group and OS + sh‐Control group. (i,j,l) ^**^ *P* < 0.01 compared with the Control group, ^##^ *P* < 0.01 compared with the AS group and AS + Vector group, and ^&&^ *P* < 0.01 compared with the AS group and AS + sh‐Control group. ALP, alkaline phosphatase; AS, adipogenic stimulation; BSPII, bone sialoprotein II; CEBP‐α, CCAAT/enhancer‐binding protein‐α; FABP4, fatty acid binding protein 4; hBMSC, human‐derived bone marrow mesenchymal stem cells; LINC00473, LINCRNA‐00473; OPN3, osteopontin 3; OS, osteogenic stimulation; PPAR‐γ, peroxisome proliferator‐activated receptor‐γ; qRT‐PCR, quantitative real‐time polymerase chain reaction; Runx‐2, runt‐related transcription factor‐2; TG, triglyceride

**FIGURE 4:**
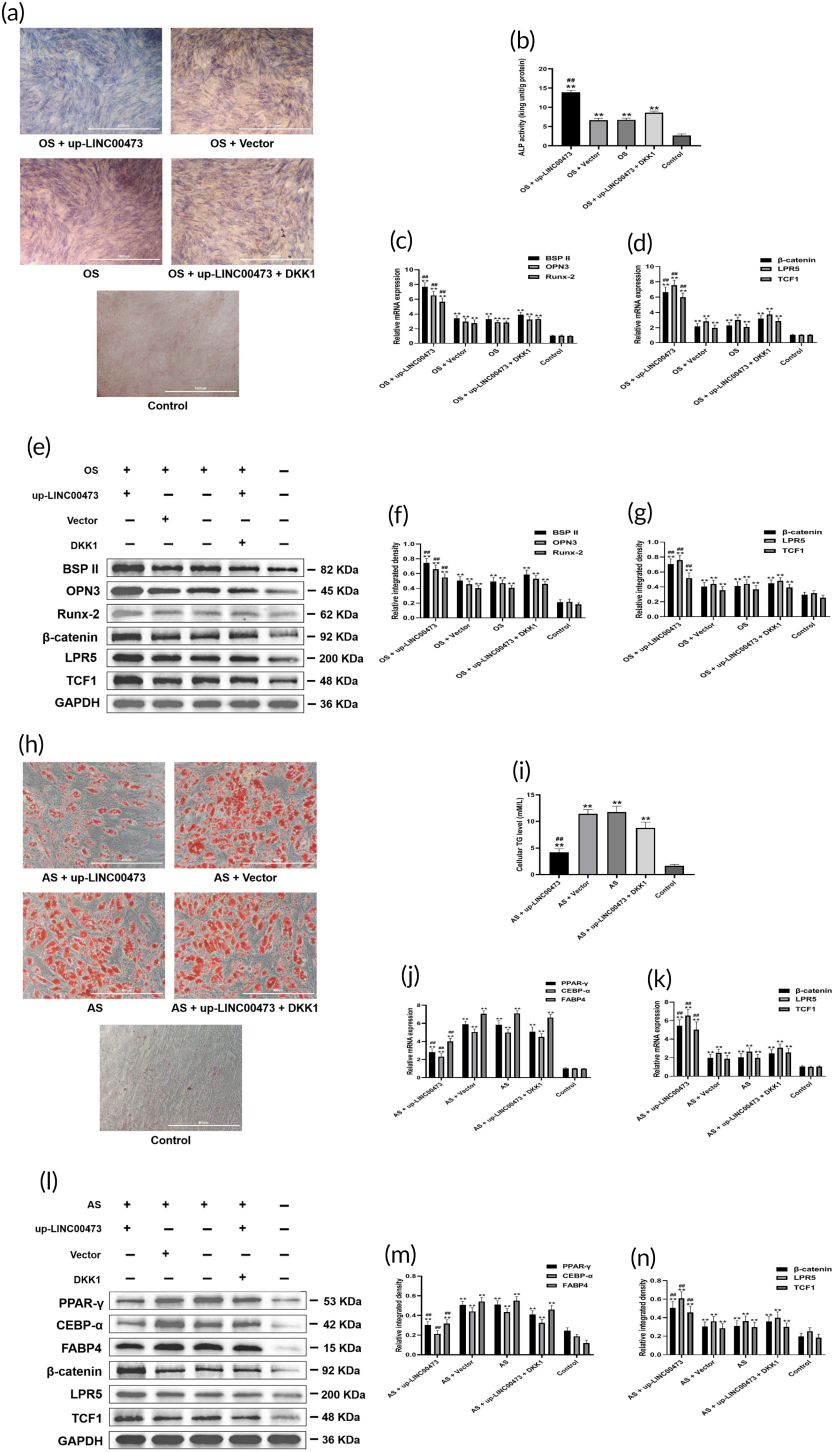
LINC00473 promoted osteogenic differentiation and suppressed the adipogenic differentiation of hBMSCs through activating the Wnt/β‐catenin signaling pathway. (a) ALP staining for osteogenic differentiation of hBMSCs under an inverted phase contrast microscope (scale bar = 1000 μm). (b) ALP activity assay for osteogenic differentiation of hBMSCs. (c) qRT‐PCR showing the mRNA expression levels of BSPII, OPN3 and Runx‐2 in each group. (d) qRT‐PCR showing the mRNA expression levels of β‐catenin, LRP5, and TCF1 in each group under the condition of osteogenic stimulation. (e) Western blotting showing the protein expression levels of BSPII, OPN3, Runx‐2, β‐catenin, LRP5, and TCF1 in each group under osteogenic stimulation. (f,g) The quantification of integrated density in target bands normalized to GAPDH. (h) Oil red O staining for adipogenic differentiation of hBMSCs under an inverted phase contrast microscope (scale bar = 400 μm). (i) Cellular TG content assay for adipogenic differentiation of hBMSCs. (j) qRT‐PCR showing the mRNA expression levels of PPAR‐γ, CEBP‐α, and FABP4 in each group. (k) qRT‐PCR showing the mRNA expression levels of β‐catenin, LRP5, and TCF1 in each group under adipogenic stimulation (AS). (l) Western blotting showing the protein expression levels of PPAR‐γ, CEBP‐α, FABP4, β‐catenin, LRP5, and TCF1 in each group under adipogenic stimulation (AS). (m,n) The quantification of integrated density in target bands normalized to GAPDH. Data are presented as mean ± standard deviation of three independent experiments. (b–d,f,g) ^**^ *P* < 0.01 compared with the Control group, and ^##^ *P* < 0.01 compared with the OS group, OS + Vector group and OS + up‐LINC00473 + DKK1 group. (i–k,m,n) ^**^ *P* < 0.01 compared with the Control group, ^##^ *P* < 0.01 compared with the the AS group, AS + Vector group and AS + up‐LINC00473 + DKK1 group. ALP, alkaline phosphatase; AS, adipogenic stimulation; BSPII, bone sialoprotein II; CEBP‐α, CCAAT/enhancer‐binding protein‐α; DKK1, Dickkopf‐related protein 1; FABP4, fatty acid binding protein 4; hBMSC, human‐derived bone marrow mesenchymal stem cells; LINC00473, LINCRNA‐00473; LRP5, low‐density lipoprotein receptor‐related protein 5; OPN3, osteopontin 3; OS, osteogenic stimulation; PPAR‐γ, peroxisome proliferator‐activated receptor‐γ; qRT‐PCR, quantitative real‐time polymerase chain reaction; Runx‐2, runt‐related transcription factor‐2; TCF1, T cell factor 1; TG, triglyceride

First, we investigated the effects of LINC00473 on osteogenic and adipogenic differentiation of hBMSCs. Recombinant lentiviruses overexpressing LINC00473 (up‐LINC00473), short‐hairpin RNAs (shRNA) against LINC00473 (sh‐LINC00473), and negative control oligonucleotides (Vector and sh‐Control) were constructed and transfected into hBMSCs (the efficiency of transfection was determined by quantitative real‐time polymerase chain reaction [qRT‐PCR] as shown in Figure 3a,b). After the hBMSCs were infected with up‐LINC00473 and sh‐LINC00473 lentivirus, respectively, green fluorescent probe was detected in more than 90% of cells (Figure 3a). In addition, the relative expression of LINC00473 in hBMSCs was significantly up‐regulated by up‐LINC00473 lentivirus (543%) and was down‐regulated by sh‐LINC00473 lentivirus (34.9%), compared to the level observed in the control group, vector group, and sh‐control group (Figure 4d). These results demonstrated successful transfection of hBMSCs with up‐LINC00473 and sh‐LINC00473 lentivirus.

Then, ALP staining, ALP activity assay, and the expression level of osteogenic markers (BSPII, OPN3, and Runx‐2) were assessed to evaluate the effect of LINC00473 on osteogenic differentiation of hBMSCs. Meanwhile, oil red O staining, cellular triglyceride (TG) levels, and the expression level of adipogenic markers (PPAR‐γ, CEBP‐α, FABP4) were used to investigate the effect of LINC00473 on adipogenic differentiation of hBMSCs.

We found that under osteogenic stimulation (OS), overexpression of LINC00473 significantly increased the number of ALP‐positive cells, the cellular ALP activity, and the protein and mRNA expression levels of BSPII, OPN3, and Runx‐2. On the contrary, the above indices were significantly decreased after LINC00473 knockdown (Figure 3c–g). Under adipogenic stimulation (AS), the overexpression of LINC00473 significantly inhibited lipid droplet formation, decreased the cellular TG levels, and down‐regulated the expression levels of PPAR‐γ, CEBP‐α, and FABP4. These effects were reversed after the knockdown of LINC00473 (Figure 3h–l).

Our results showed that the overexpression of LINC00473 could promote osteogenic differentiation and suppress adipogenic differentiation of hBMSCs, while LINC00473 knockdown had exactly opposite effects.

### 2.4 LINC00473 promoted osteogenic differentiation and suppressed the adipogenic differentiation of hBMSCs through activating the Wnt/β‐catenin signaling pathway

Subsequently, a blocking experiment was designed to investigate the role of the Wnt/β‐catenin signaling pathway in the regulation of LINC00473 on osteogenic and adipogenic differentiation of hBMSCs. We first confirmed that the overexpression of LINC00473 exerted the same effects above described (Figure 4a,b,h–i). Western blotting and qRT‐PCR analyses showed that the protein and mRNA expression levels of LRP5, β‐catenin, and TCF1 were significantly up‐regulated, indicating the activation of the Wnt/β‐catenin signaling pathway under the condition of OS (Figure 4d,e,g) or AS (Figure 4k,l,n).

Notably, DKK1 (a specific inhibitor of Wnt/β‐catenin signaling pathway) partially reversed the effects of LINC00473 on ALP activity, expression levels of BSPII, OPN3 and Runx‐2 (Figure 4a–c,e,f), lipid droplet formation, cellular TG levels and the expression levels of PPAR‐γ, CEBP‐α, and FABP4 in hBMSCs (Figure 4h–j,l,m). Moreover, the regulation of LINC00473 on LRP5, β‐catenin, and TCF1 were also antagonized by DKK1 (Figure 4d,g,k,n).

Our results showed that LINC00473 promoted osteogenic differentiation and suppressed the adipogenic differentiation of hBMSCs by activating the Wnt/β‐catenin signaling pathway.

### 2.5 miR‐23a‐3p is a key regulator of the LINC00473‐activated LRP5/Wnt/β‐catenin signaling pathway in hBMSCs

**FIGURE 5:**
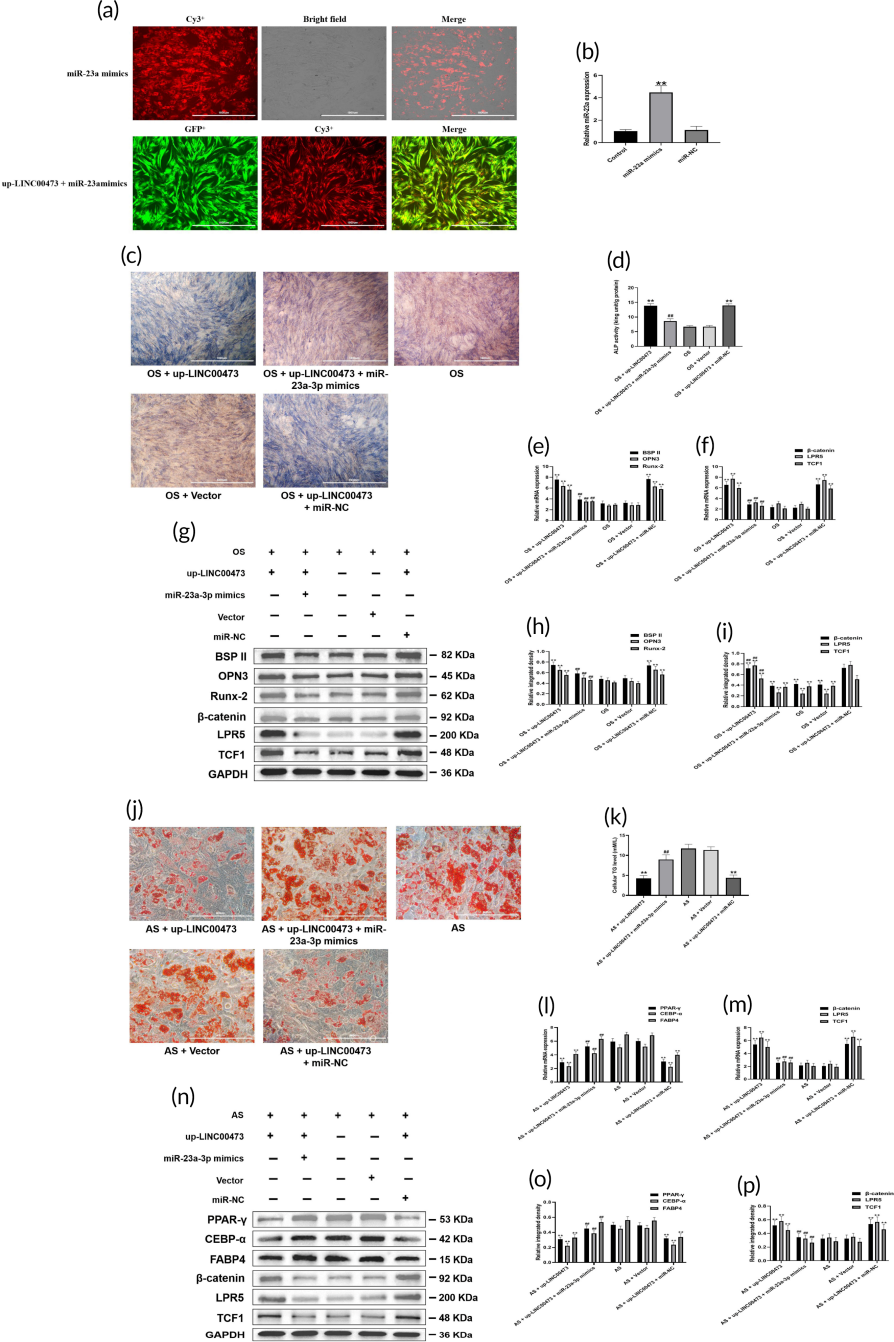
miR‐23a‐3p mediated LINC00473‐activated LRP5/Wnt/β‐catenin signaling pathway axis in the osteogenic and adipogenic differentiation of hBMSCs. (a) Efficiency of transfection in hBMSCs for miR‐23a‐3p and LINC00473 under a fluorescence microscope (scale bar = 1000 μm). (b) Efficiency of transfection in hBMSCs for miR‐23a‐3p determined by qRT‐PCR analysis. (c) ALP staining for osteogenic differentiation of hBMSCs under an inverted phase contrast microscope (scale bar = 1000 μm). (d) ALP activity assay for osteogenic differentiation of hBMSCs. (e) qRT‐PCR showing the mRNA expression levels of BSPII, OPN3, and Runx‐2 in each group. (f) qRT‐PCR showing the mRNA expression levels of β‐catenin, LRP5, and TCF1 in each group under osteogenic stimulation. (g) Western blotting showing the protein expression levels of BSPII, OPN3, Runx‐2, β‐catenin, LRP5, and TCF1 in each group under osteogenic stimulation. (h,i) The quantification of integrated density in target bands normalized to GAPDH. (j) Oil red O staining for adipogenic differentiation of hBMSCs under an inverted phase contrast microscope (scale bar = 400 μm). (k) Cellular TG content assay for adipogenic differentiation of hBMSCs. (l) qRT‐PCR showing the mRNA expression levels of PPAR‐γ, CEBP‐α, and FABP4 in each group. (m) qRT‐PCR showing the mRNA expression levels of β‐catenin, LRP5, and TCF1 in each group under adipogenic stimulation (AS). (n) Western blotting showing the protein expression levels of PPAR‐γ, CEBP‐α, FABP4, β‐catenin, LRP5, and TCF1 in each group under adipogenic stimulation (AS). (o,p) The quantification of integrated density in target bands normalized to GAPDH. Data are presented as mean ± standard deviation of three independent experiments. (b) ^**^ *P* < 0.01 compared with the Control group and miR‐NC group. (d–f,h,i) ^**^ *P* < 0.01 compared with the OS group and OS + Vector group, and ^##^ *P* < 0.01 compared with the OS + up‐LINC00473 group and OS + up‐LINC00473 + miR‐NC group. (k–m,o,p) ^**^ *P* < 0.01 compared with the AS group and AS + Vector group, and ^##^ *P* < 0.01 compared with the AS + up‐LINC00473 group and AS + up‐LINC00473 + miR‐NC group. ALP, alkaline phosphatase; AS, adipogenic stimulation; BSPII, bone sialoprotein II; CEBP‐α, CCAAT/enhancer‐binding protein‐α; DKK1, Dickkopf‐related protein 1; FABP4, fatty acid binding protein 4; hBMSC, human‐derived bone marrow mesenchymal stem cells; LINC00473, INCRNA‐00473; LRP5, low‐density lipoprotein receptor‐related protein 5; miR‐23a‐3p, MicroRNA‐23a‐3p; OPN3, osteopontin 3; OS, osteogenic stimulation; PPAR‐γ, peroxisome proliferator‐activated receptor‐γ; qRT‐PCR, quantitative real‐time polymerase chain reaction; Runx‐2, runt‐related transcription factor‐2; TCF1, T cell factor 1; TG, triglyceride

hBMSCs cells were co‐transfected with miR‐23a‐3p mimics and recombinant lentiviruses overexpressing LINC00473 to investigate the underlying mechanism of LINC00473 on the regulation of the Wnt/β‐catenin signaling pathway. The efficiency of transfection was determined by qRT‐PCR. After the hBMSCs were transfected with miR‐23a‐3p mimics, red fluorescent with Cy3 was detected in more than 90% of cells (Figure 5a). In addition, the relative expression of miR‐23a‐3p in hBMSCs was significantly up‐regulated by miR‐23a‐3p mimics (448%), compared to the level observed in the control group and miR‐NC group (Figure 5b). These results demonstrated successful transfection of hBMSCs with miR‐23a‐3p mimics.

The effects of the overexpression of LINC00473 were further confirmed in OS (Figure 5c–e,g,h) and AS (Figure 5j–l,n,o) of hBMSCs cells, as well as on the activation of the Wnt/β‐catenin signaling pathway (Figure 5f,i,m,p). Interestingly, the transfection of miR‐23a‐3p mimics inhibited the effects induced by LINC00473 overexpression (Figure 5c–p).

Due to that the targeted binding between miR‐23a‐3p and LRP5 had been confirmed in our previous study,^38^ the above data indicated that miR‐23a‐3p regulated the LINC00473‐activated LRP5/Wnt/β‐catenin signaling pathway axis in hBMSCs cells.

### 2.6 miR‐23a‐3p is involved in LINC00473‐activated PEBP1/Akt/Bad/Bcl‐2 signaling pathway axis in Dex‐induced apoptosis of hBMSCs

**FIGURE 6:**
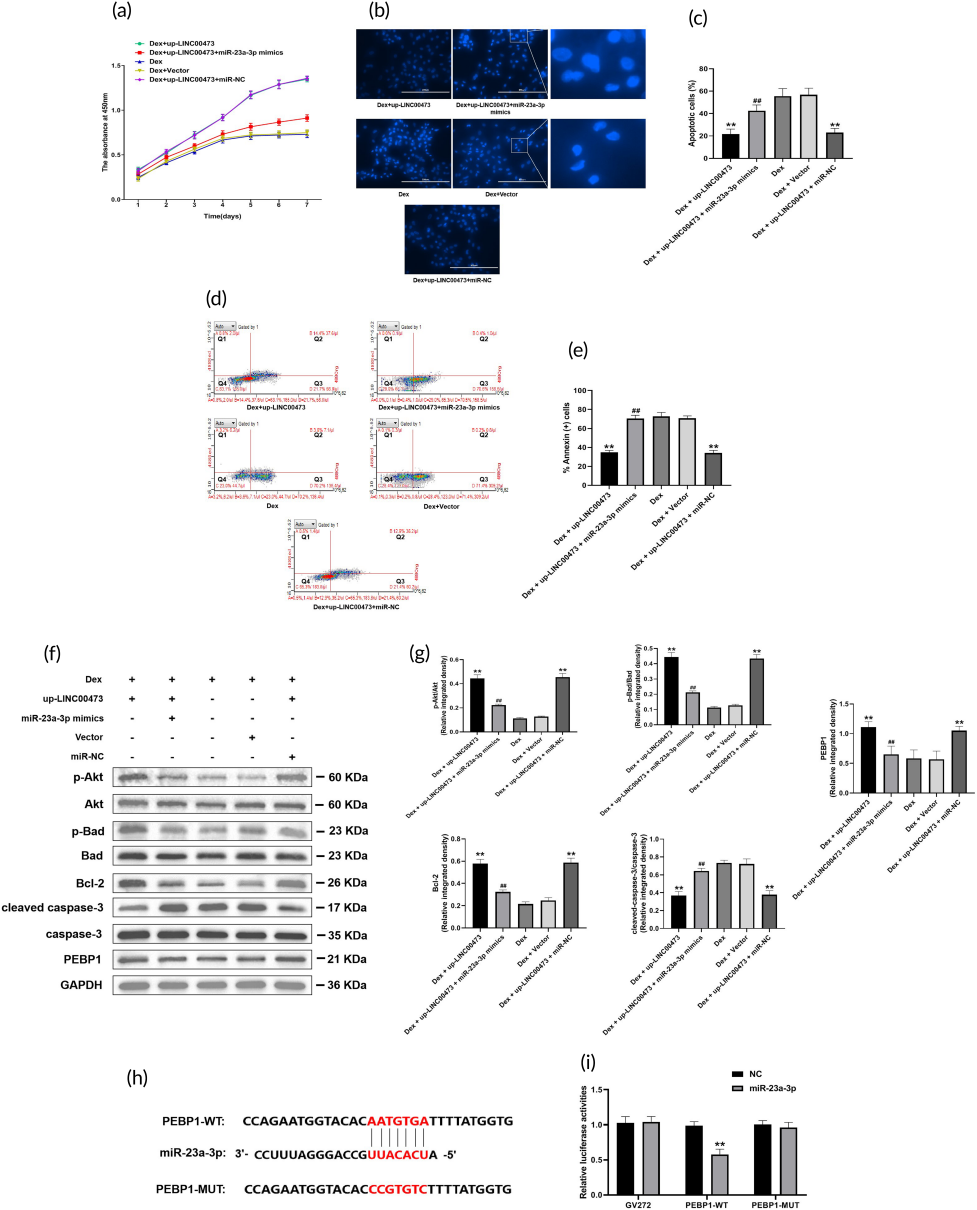
miR‐23a‐3p is involved in LINC00473‐activated PEBP1/Akt/Bad/Bcl‐2 signaling pathway axis in Dex‐induced apoptosis of hBMSCs. (a) CCK‐8 assay showing the proliferation viability of hBMSCs in each group. (b) Hoechst 33342 staining showing the morphology of apoptotic cells in each group under a fluorescence microscope (scale bar = 200 μm). (c) Cell count of the percentage of apoptotic cells in each group. Six randomly selected fields were quantified, and a total of three hundred cells in each group were counted. (d) Flow cytometry of apoptotic cells with Annexin V‐PE and 7‐AAD staining: Q1 showing the percentage of necrotic cells, Q2 showing the percentage of late apoptotic cells, Q3 showing the percentage of early apoptotic cell, Q4 showing the percentage of normal cells. (e) The percentage of Annexin^+^ cells in Q2 and Q3 in each group. (f) Western blotting showing the protein expression of p‐Akt, Akt, P‐Bad, Bad, Bcl‐2, cleaved‐caspase‐3, caspase‐3, and PEBP1 in each group. (g) The quantification of integrated density in target bands normalized to GAPDH. (h) The sequences of wild‐type and mutant‐type PEBP1 without miR‐23a‐3p‐binding sites. (i) Relative luciferase activities of wild‐type or mutant‐type PEBP1 co‐transfected with miR‐23‐3p mimics. Data are presented as mean ± standard deviation of three independent experiments. (c,e,g) ^**^ *P* < 0.01 compared with the Dex group and the Dex + Vector group, ^##^ *P* < 0.01 compared with the Dex + up‐LINC00473 group and Dex + up‐LINC00473 + miR‐NC group. (i) ^**^ *P* < 0.01 compared with the GV272 + miR‐23a‐3p group and PEBP1‐MUT + miR‐23a‐3p group. Akt, protein kinase B; Bad, Bcl‐2 associated death promoter; Bcl‐2, B‐cell lymphoma 2; CCK‐8, Cell Counting Kit‐8; Dex, dexamethasone; hBMSCs, human‐derived bone marrow mesenchymal stem cells; LINC00473, LINCRNA‐00473; miR‐23a‐3p, MicroRNA‐23a‐3p; p‐Akt, phosphorylated protein kinase B; p‐Bad, phosphorylated Bcl‐2 associated death promoter; Q, quadrant

Based on our previous study, LINC00473 could rescue hBMSCs from Dex‐induced apoptosis through the activation of the PEBP1/Akt/Bad/Bcl‐2 signaling pathway axis.^41^ Moreover, the previous predictions indicated that there were target sites between miR‐23a‐3p and LINC00473, as well as miR‐23a‐3p and PEBP1. Therefore, miR‐23a‐3p could act as a bridge to connect LINC00473 and PEBP1. In the present study, we used 10^−6^ mol/L Dex to induce the apoptosis of hBMSCs, and employed recombinant lentiviruses overexpressing LINC00473 to transfect hBMSCs. Subsequently, cell proliferation from day 1 to 7 was evaluated by the CCK‐8 assay, and cell apoptosis was observed by Hoechst 33342 staining and flow cytometry assay for Annexin V‐PE/7‐AAD double‐staining. As previously reported,^41^ LINC00473 protected hBMSCs cells from Dex‐induced apoptosis; an effect mediated by the activation of the PEBP1/Akt/Bad/Bcl‐2 signaling pathway axis. Specifically, in the presence of Dex, overexpression of LINC00473 could promote the proliferation of hBMSCs (Figure 6a), decrease the number of apoptotic cells (Figure 6b–e), and inhibit the caspase‐3 activity, while promoting the phosphorylation of Akt and Bad and increasing the protein expression of PEBP1 and Bcl‐2 (Figure 6f,g). Notably, the anti‐apoptotic effect of LINC00473 and the corresponding activation of the PEBP1/Akt/Bad/Bcl‐2 signaling pathway axis were suppressed dramatically by the transfection of miR‐23a‐3p mimics (Figure 6a–g).

Furthermore, the interactions between miR‐23a‐3p and PEBP1 were verified by a dual‐luciferase reporter assay system. The plasmid‐PEBP1 vectors were constructed by inserting the sequences of wild‐ and mutant‐type (without miR‐23a‐3p‐binding sites) PEBP1 upstream of the luciferase gene (Figure 6h). We found that miR‐23a‐3p mimics significantly decreased the luciferase activity of wild‐type PEBP1 but not mutant‐type PEBP1 or empty vector in hBMSCs, while miR‐NC had no effect (Figure 6i), showing that miR‐23a‐3p could bind to PEBP1 directly.

Our data demonstrated that miR‐23a‐3p was involved in LINC00473‐activated PEBP1/Akt/Bad/Bcl‐2 signaling in hBSMCs exposed to Dex.

### 2.7 LINC00473‐targeted miR‐23a‐3p as a ceRNA

According to our previous results of fluorescent in situ hybridization (FISH),^40^ LINC00473 was located in both the cytoplasm and nucleus of hBMSCs, suggesting that it induce a post‐transcriptional process. Considering that numerous lncRNAs act as ceRNAs to regulate the distribution of miRNA, we explored whether there was a similar interaction between LINC00473 and miR‐23a‐3p.

**FIGURE 7:**
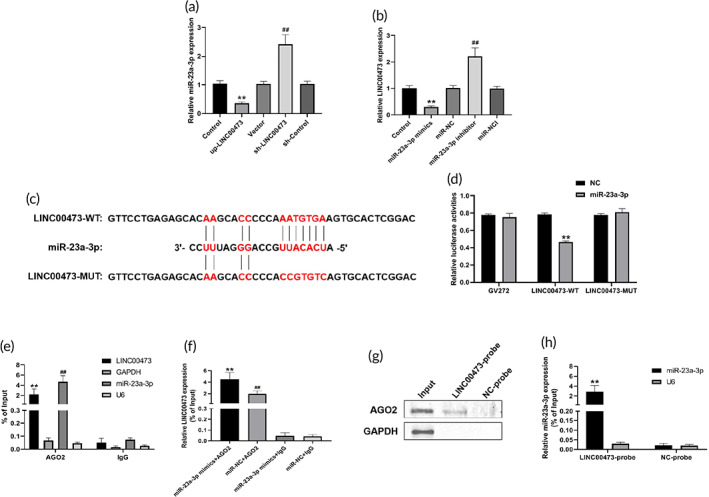
LINC00473‐targeted miR‐23a‐3p as a ceRNA. (a) qRT‐PCR analysis for the regulation of LINC00473 on miR‐23a‐3p. (b) qRT‐PCR analysis for the regulation of miR‐23a‐3p on LINC00473. (c) The sequences of wild‐type and mutant‐type LINC00473 without miR‐23a‐3p‐binding sites. (d) Relative luciferase activities of wild‐type or mutant‐type LINC00473 co‐transfected with miR‐23‐3p mimics. (e) qRT‐PCR showing enrichment efficiency of LINC00473 and miR‐23a‐3p based on AGO2‐RIP. (f) qRT‐PCR showing enrichment efficiency of LINC00473 after transfection with miR‐23a‐3p mimics or miR‐NC based on AGO2‐RIP. (g) Western blotting showing the protein expression of AGO2 in each group based on RAP. (h) qRT‐PCR showing the mRNA expression of miR‐23a‐3p in each group based on RAP. Data are presented as mean value ± standard deviation of three independent experiments. (a) ^**^ *P* < 0.01 compared with the Control group and Vector group, ^##^ *P* < 0.01 compared with the Control group and sh‐Control group. (b) ^**^ *P* < 0.01 compared with the Control group and miR‐NC group, ^##^ *P* < 0.01 compared with the Control group and miR‐NCI group. (d) ^**^ *P* < 0.01 compared with the GV272 + miR‐23a‐3p group and LINC00473‐MUT + miR‐23a‐3p group. (e) ^**^ *P* < 0.01 compared with the LINC00473 + IgG group, ^##^ *P* < 0.01 compared with the miR‐23a‐3p + IgG group. (f) ^**^ *P* < 0.01 compared with the miR‐23a‐3p mimics + IgG group and miR‐NC + AGO2 group, ^##^ *P* < 0.01 compared with the miR‐NC + IgG group. (h) ^**^ *P* < 0.01 compared with NC‐probe group. AGO2, Argonaute 2; ceRNA, competing endogenous RNA; LINC00473, LINCRNA‐00473; miR‐23a‐3p, MicroRNA‐23a‐3p; qRT‐PCR, quantitative real‐time polymerase chain reaction; RAP, RNA antisense purification; RIP, RNA immunoprecipitation

First, we investigated the mutual regulation between LINC00473 and miR‐23a‐3p. qRT‐PCR analysis showed that overexpression of LINC00473 decreased the enrichment of miR‐23a‐3p, and knockdown of LINC00473 significantly increased the expression of miR‐23a‐3p in hBMSCs. Furthermore, miR‐23a‐3p mimics down‐regulated the expression of LINC00473, and miR‐23a‐3p inhibitors up‐regulated the expression of LINC00473 in hBMSCs. These data revealed that there was a negative interaction between LINC00473 and miR‐23a‐3p in hBMSCs (Figure 7a,b).

We employed a dual‐luciferase reporter assay system to verify the interactions between LINC00473 and miR‐23a‐3p. The plasmid‐LINC00473 vectors were constructed by inserting the sequences of wild‐ and mutant‐type (without miR‐23a‐3p‐binding sites) LINC00473 downstream of the luciferase gene (Figure 7c). We found that miR‐23a‐3p mimics significantly decreased the luciferase activity of wild‐type LINC00473 but not mutant‐type LINC00473 or empty vector in hBMSCs, while miR‐NC had no effect (Figure 7d). These data demonstrate that miR‐23a‐3p could bind to LINC00473.

It was shown that the protein AGO2 could bind to miRNAs and mRNAs and regulate the abundance of miRNAs in post‐transcription, thereby affecting the biological function of miRNAs.^47^, ^48^, ^49^ Therefore, to investigate whether AGO2 was involved in the modulation of miR‐34a‐5p by LINC00473, we performed AGO2‐related RNA immunoprecipitation (AGO2‐RIP) to pull down endogenous miRNAs and lncRNAs bound to AGO2 and detected the expression of LINC00473 and miR‐23a‐3p in the pull‐down complex by qRT‐PCR. Our results showed that endogenous LINC00473 and miR‐23a‐3p were specifically enriched by the AGO2 protein in hBMSCs, but not by a control IgG protein (Figure 7e). Moreover, we conducted AGO2‐RIP in hBMSCs transfected with miR‐23a‐3p mimics. Surprisingly, the enrichment of endogenous LINC00473 pull‐down by AGO2 was increased in hBMSCs transfected with miR‐23a‐3p mimics compared with that of miR‐NC (Figure 7F).

We also performed RNA antisense purification (RAP) to further confirm the specific binding between LINC00473, miR‐23a‐3p, and AGO2. The 5'‐biotinylated LINC00473 probes were designed and used in the RAP experiment, and the protein expression of AGO2 and gene expression of miR‐23a‐3p in the pull‐down complex were assessed. As expected, our data indicated that both AGO2 protein and miR‐23a‐3p were specifically enriched by LINC00473 probes, but not by NC‐probes (Figure 7g,h). Taken together, these findings revealed the interaction between LINC00473 and miR‐23a‐3p in an AGO2‐dependent manner.

### 2.8 LINC00473 promoted osteogenic differentiation and cell migration, and suppressed the adipogenic differentiation and Dex‐induced apoptosis of rBMSCs

In consideration of our previous study,^40^ indicating that LINC00473 was one of the differentially expressed genes between BMSCs from patients with SONFH and control patients with femoral neck fracture, we intended to perform LINC00473‐modified rBMSCs transplantation into rats with SONFH to further reveal the biological function of LINC00473 in vivo. Notably, in vivo experiment for LINC00473 in mice has been reported,^50^ although LINC00473 was a primate‐specific lncRNA and not normally expressed in rodents.^51^ In addition, there was no lack of using rodents to reveal the biological function in vivo for primate‐specific lncRNAs.^52^

**FIGURE 8:**
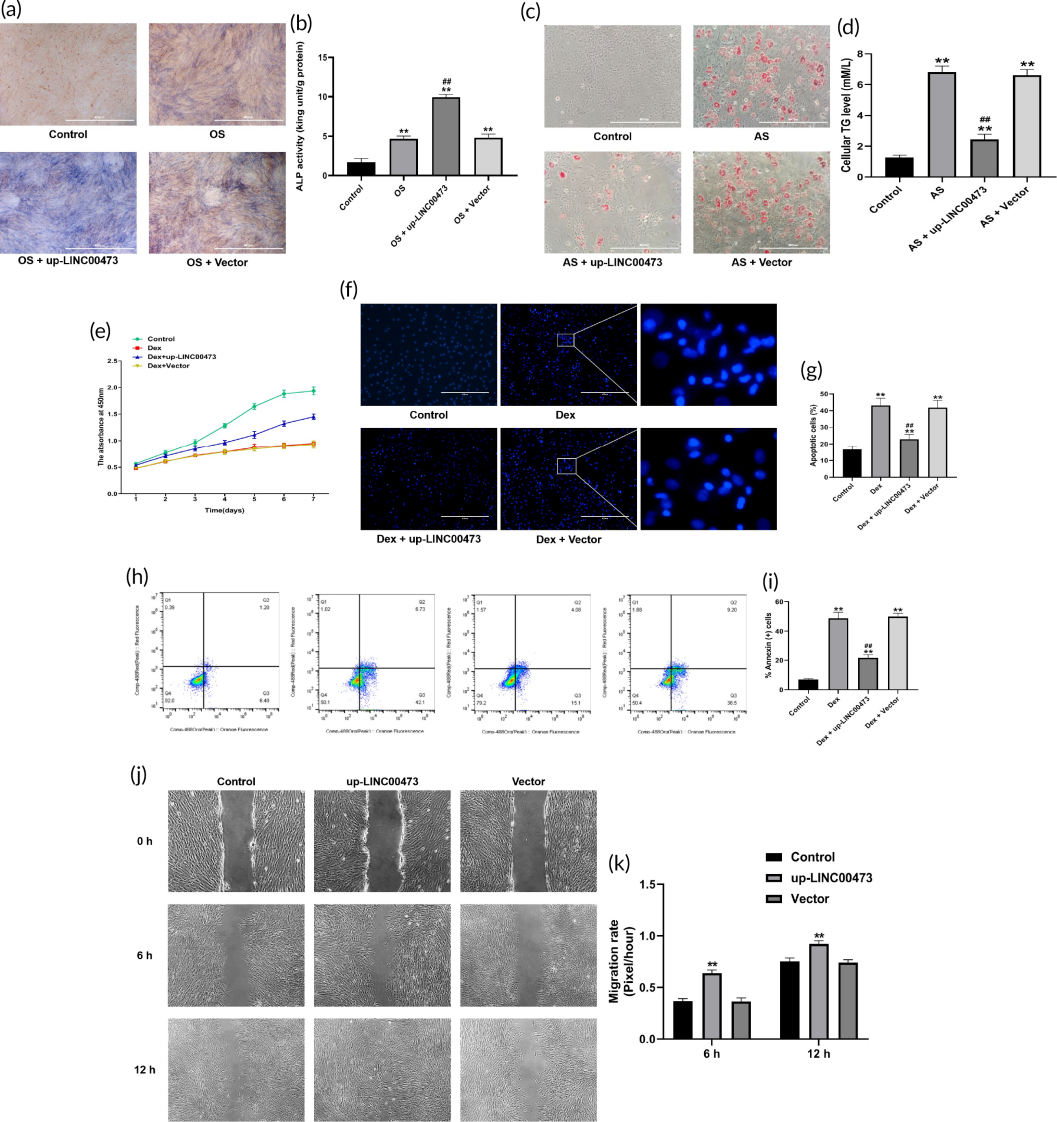
The effects of LINC00473 on the osteogenic, adipogenic differentiation, viability, and migration of rBMSCs. (a) ALP staining for osteogenic differentiation of rBMSCs under an inverted phase contrast microscope (scale bar = 400 μm). (b) ALP activity assay for osteogenic differentiation of rBMSCs. (c) Oil red O staining for adipogenic differentiation of rBMSCs under an inverted phase contrast microscope (scale bar = 400 μm). (d) Cellular TG content assay for adipogenic differentiation of rBMSCs. (e) CCK‐8 assay showing the proliferation viability of rBMSCs in each group. (f) Hoechst 33342 staining showing the morphology of apoptotic cells in each group under a fluorescence microscope (scale bar = 400 μm). (g) Cell count of the percentage of apoptotic cells in each group. Six randomly selected fields were quantified, and a total of three hundred cells in each group were counted. (h) Flow cytometry of apoptotic cells with Annexin V‐PE and 7‐AAD staining: Q1 showing the percentage of necrotic cells, Q2 showing the percentage of late apoptotic cells, Q3 showing the percentage of early apoptotic cell, Q4 showing the percentage of normal cells. (i) The percentage of Annexin^+^ cells in Q2 and Q3 in each group. (j) Representative images for scratch wound‐healing assay under an inverted phase contrast microscope captured at 0, 6 and 12 h. (k) Migration rate in each group. Data are presented as mean ± standard deviation of three independent experiments. (b) ^**^ *P* < 0.01 compared with the Control group, and ^##^ *P* < 0.01 compared with the OS group and OS + Vector group. (d) ^**^ *P* < 0.01 compared with the Control group, ^##^ *P* < 0.01 compared with the AS group and AS + Vector group. (g,i) ^**^ *P* < 0.01 compared with the Control group, ^##^ *P* < 0.01 compared with the Dex group and and the Dex + Vector group. (k) ^**^ *P* < 0.01 compared with the Control group and Vector group. ALP, alkaline phosphatase; AS, adipogenic stimulation; CCK‐8, Cell Counting Kit‐8; Dex, dexamethasone; LINC00473, LINCRNA‐00473; OS, osteogenic stimulation; Q, quadrant; rBMSC, rat‐derived bone marrow mesenchymal stem cells; TG, triglyceride

Therefore, we first investigated the effect of LINC00473 on osteogenic and adipogenic differentiation of rBMSCs. Our finding showed that overexpression of LINC00473 significantly increased the number of ALP‐positive cells and the cellular ALP activity while decreased the lipid droplet formation and the cellular TG levels in rBMSCs (Figure 8a–d), indicating that overexpression of LINC00473 could promote osteogenic differentiation and suppress the adipogenic differentiation of rBMSCs. In addition, the effect of LINC00473 on the Dex‐induced apoptosis of rBMSCs was observed by Hoechst 33342 staining, flow cytometry assay, and CCK‐8 assay. We found that overexpression of LINC00473 could rescue hBMSCs from Dex‐induced apoptosis (Figure 8e–i). Interestingly, these results were in line with our findings in hBMSCs.^39^ Furthermore, we performed a scratch wound healing assay to observe the effect of LINC00473 on rBMSCs migration. As expected, the rBMSCs overexpressing LINC00473 migrated and recovered into the denuded area in a shorter time compared with control cells (Figure 8j,k), suggesting that overexpression of LINC00473 could enhance the migration of rBMSCs in vitro. The above data demonstrated that overexpression of LINC00473 in rBMSCs had similar effects to those observed in hBMSCs cells.

### 2.9 PLGA hydrogel provided a suitable environment for harboring rBMSCs

**FIGURE 9:**
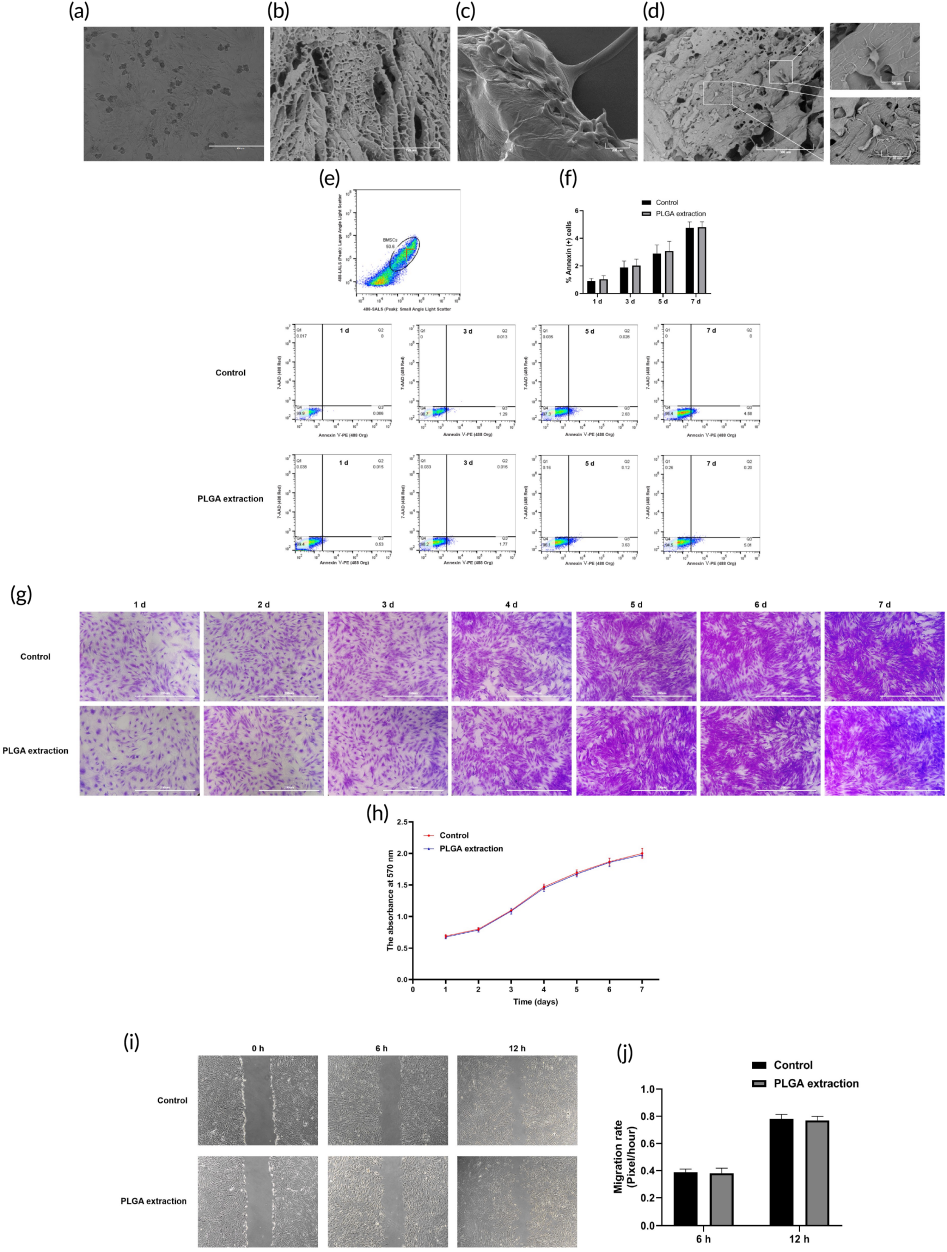
PLGA hydrogel can provide a suitable environment for harboring rBMSCs. (a) Representative images showing the morphology of rBMSCs on the surface of a PLGA hydrogel under an inverted phase contrast microscope (scale bar = 400 μm). (b) Representative SEM images of the PLGA hydrogel structure. (c,d) Representative SEM images of rBMSCs encapsulated in a PLGA hydrogel after 7 days of culture. (e) Flow cytometry of apoptotic cells with Annexin V‐PE and 7‐AAD staining: Q1 showing the percentage of necrotic cells, Q2 showing the percentage of late apoptotic cells, Q3 showing the percentage of early apoptotic cell, Q4 showing the percentage of normal cells. (f) The percentage of Annexin^+^ cells in Q2 and Q3 in each group. (g) Crystal violet assay showing the proliferation viability of rBMSCs continuously treated by the PLGA hydrogels maceration extract for 7 days. (h) The quantification of crystal violet assay using the absorbance detected by a microplate reader at 570 nm. (i) Representative images for scratch wound‐healing assay under an inverted phase contrast microscope captured at 0, 6 and 12 h. (j) Migration rate in each group. Data are presented as mean value ± standard deviation of three independent experiments. PLGA, polylactic‐co‐glycolic acid; Q, quadrant; rBMSCs, rat‐derived bone marrow mesenchymal stem cells; SEM, scanning electron microscope

Stem cell transplantation combined with tissue engineering has shown a promising prospect in the treatment of SONFH.^44^, ^53^ Injectable thermosensitive PLGA hydrogel, a biodegradable synthetic copolymer, was used as a cell‐delivery carrier in cell transplantation experiments, based on previous reports.^45^, ^46^ First, we evaluated the cytocompatibility of PLGA hydrogel for rBMSCs. The morphology of rBMSCs on the surface of the PLGA hydrogel was observed by an inverted phase contrast microscope. We found that rBMSCs cultured on the surface of PLGA hydrogels for 3 days showed a fusiform fibroblast‐like morphology (Figure 9a), which was similar to that of native rBMSCs. In addition, the morphology of rBMSCs encapsulated within the PLGA hydrogels was investigated under a scanning electron microscope (SEM). Injectable thermosensitive PLGA hydrogels showed a large number of discontinuous 20–50 μm pores, which were interconnected to form a three‐dimensional network structure with a honeycomb appearance (Figure 9b). After 7 days of culture, rBMSCs began to expand, and adhered to the pore wall as pseudopodium (Figure 9c,d).

We further evaluated the toxicity of PLGA hydrogel on rBMSCs. The effects of a PLGA hydrogel maceration extract on the proliferation and apoptosis of rBMSCs were evaluated by crystal violet and flow cytometry assay, respectively. Continuous treatment with the PLGA hydrogels maceration extract for 7 days did not affect the cell proliferation nor did it induce apoptosis of rBMSCs cells (Figure 9e–h). In addition, the role of PLGA hydrogel maceration extract on the migration of rBMSCs in vitro were assessed by a scratch wound healing assay. Treatment with the PLGA hydrogels maceration extract for 6 and 12 h had no effects on the migration of rBMSCs (Figure 9i,j).

These results indicated that PLGA hydrogels showed a reliable cell biocompatibility and could provide a suitable environment for harboring rBMSCs.

### 2.10 Transplantation of PLGA hydrogels loaded with rBMSCs overexpressing LINC00473 in SONFH rats

**FIGURE 10:**
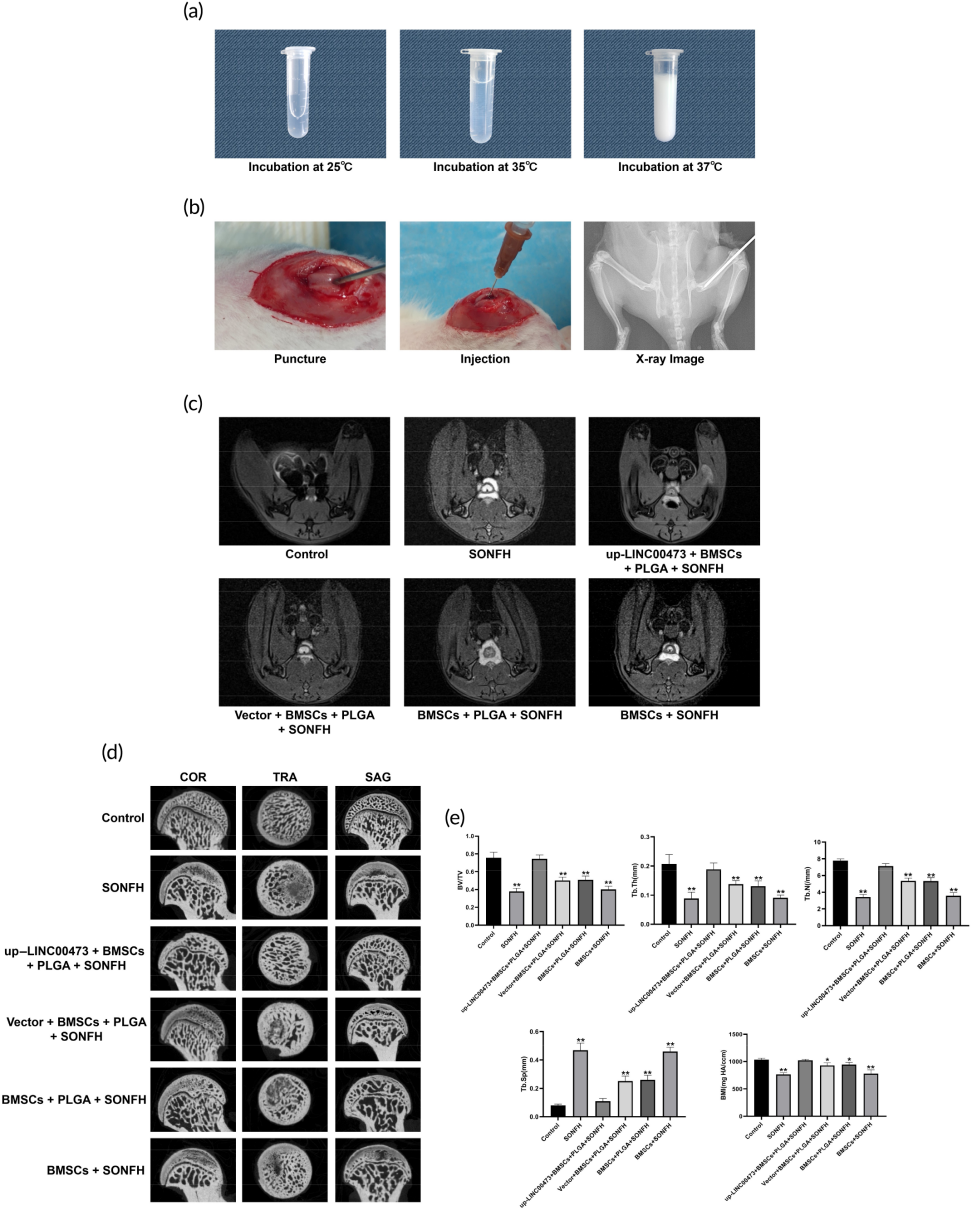
Imaging of the transplantation of PLGA hydrogels loaded with rBMSCs overexpressing LINC00473 in the treatment of SONFH in rats. (a) Status of thermosensitive PLGA hydrogels at various temperatures. (b) Representative images showing the details of the transplantation operation. (c) Representative MRI images showing the signal of the femoral head of rats in each group after treatment for 12 weeks. (d) Representative micro‐CT images showing the subchondral bone of the femoral head of rats in each group after treatment for 12 weeks. (e) Qualitative analyzes for micro‐CT scanning. Data are presented as mean value ± standard deviation. ^*^ *P* < 0.05 compared with the Control group and up‐LINC00473 + BMSCs + PLGA + SONFH group, and ^**^ *P* < 0.01 compared with the Control group and up‐LINC00473 + BMSCs + PLGA + SONFH group. LINC00473, LINCRNA‐00473; MRI, magnetic resonance imaging; PLGA, polylactic‐co‐glycolic acid; rBMSCs, rat‐derived bone marrow mesenchymal stem cells; SONFH, Steroid‐induced osteonecrosis of the femoral head

To further explore the role of LINC00473 in the treatment of SONFH, a rat model of SONFH was developed by intramuscular injection of methylprednisolone (MPS) (SONFH group), followed by femoral intramedullary transplantation of PLGA hydrogels loaded with rBMSCs overexpressing LINC00473 (up‐LINC00473 + BMSCs + PLGA + SONFH group), or transplantation of PLGA hydrogels loaded with rBMSCs transfected with a control vector (Vector + BMSCs + PLGA + SONFH group), or transplantation of PLGA hydrogels loaded with rBMSCs (BMSCs + PLGA + SONFH group), or transplantation of rBMSCs (BMSCs + SONFH group). In addition, healthy rats were used as control (Control group). As shown in Figure 10a, thermosensitive PLGA hydrogels had free flowing liquid at 25°C, but gradually converted to gel at 35–37°C. This characteristic of thermosensitive PLGA hydrogels facilitated an intraoperative injection for BMSCs transplantation. The details of the operation are shown in Figure 10b.

After treatment for 12 weeks, T_2_‐weighed magnetic resonance imaging (MRI) showed a subchondral abnormally high signal (a manifestation of bone marrow edema and osteonecrosis) in the femoral head of rats in the SONFH group, indicating that these rats had successfully developed SONFH. Surprisingly, a normal subchondral signal was found in the femoral head of rats in the up‐LINC00473 + BMSCs + PLGA + SONFH group, while a mildly elevated subchondral signal was found in the femoral head of rats of the Vector + BMSCs + PLGA + SONFH group and BMSCs + PLGA + SONFH group. However, a remarkably high subchondral signal was found in the femoral head of rats in the BMSCs + SONFH group (Figure10c). These data showed that transplantation of PLGA hydrogels loaded with rBMSCs overexpressing LINC00473 could relieve the bone marrow edema and osteonecrosis induced by glucocorticoid injection in rats.

Furthermore, micro‐CT scanning was performed to evaluate the subchondral bone of the femoral head. Typical representations of osteonecrosis, such as trabecular resorption, bone mineral loss, and cystic degeneration, were found in the subchondral area of the femoral head of rats in the SONFH and BMSCs + SONFH groups. In contrast, these subchondral changes were less pronounced in rats from the Vector + BMSCs + PLGA + SONFH and BMSCs + PLGA + SONFH groups. Importantly, intact and well‐distributed subchondral trabeculae were observed in up‐LINC00473 + BMSCs + PLGA + SONFH rats (Figure 10d). Further qualitative analyses showed that, in addition to increased trabecular separation (Tb.Sp), other microstructural parameters, such as bone volume per tissue volume (BV/TV), trabecular thickness (Tb.Th), and trabecular number (Tb.N), were significantly decreased in rats from the SONFH and BMSCs + SONFH groups. However, these parameters were reversed in rats from the up‐LINC00473 + BMSCs + PLGA + SONFH group (Figure 10e).

**FIGURE 11:**
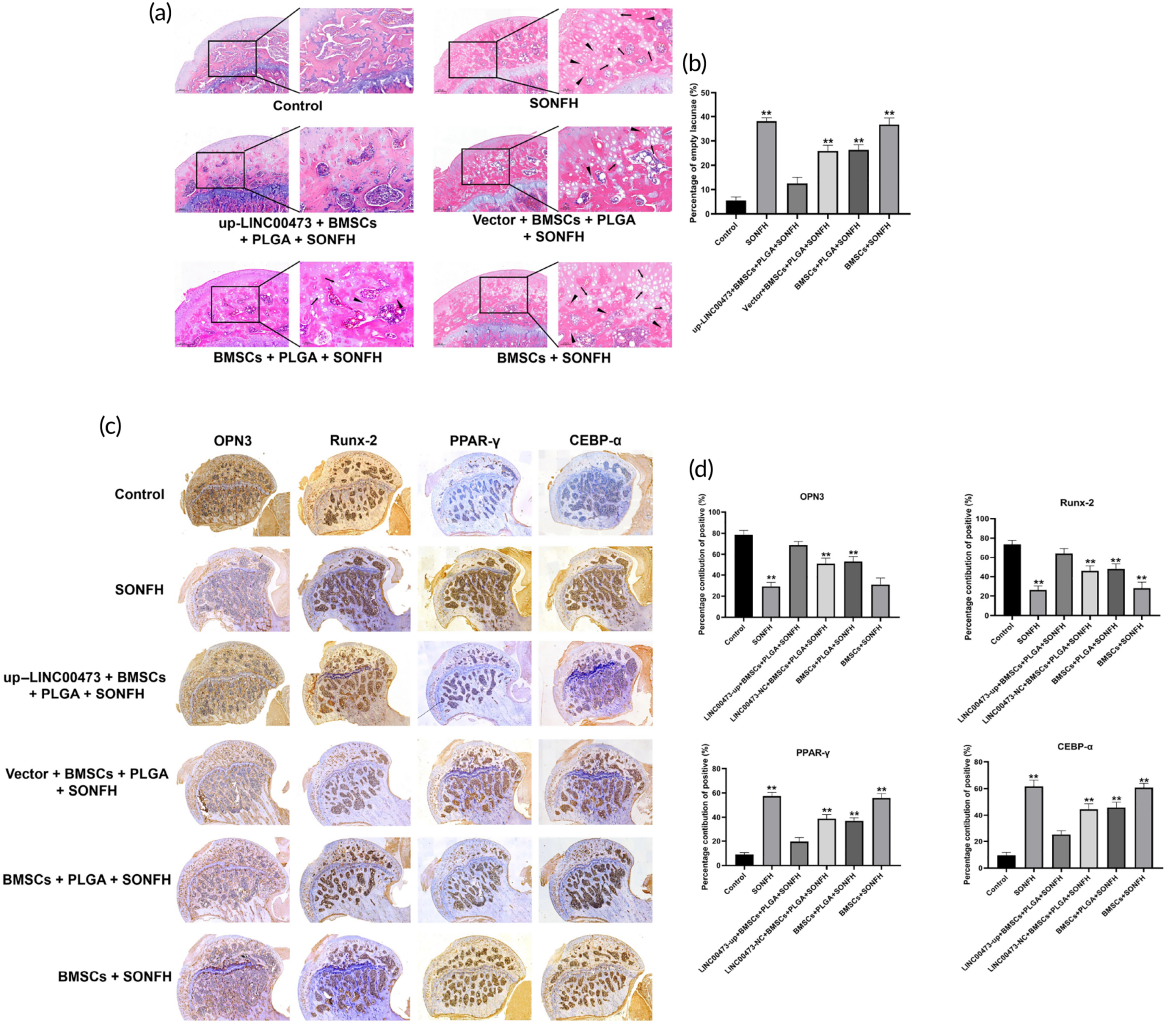
Histological analysis of the transplantation of PLGA hydrogels loaded with rBMSCs overexpressing LINC00473 in SONFH rats. (a) Representative images showing HE staining of the subchondral bone of the femoral head of rats in each group after treatment for 12 weeks. (b) Percentage of empty lacunae in the bone trabecula of the femoral head in each group after treatment for 12 weeks. (c) Representative images showing IHC for the expression of PPAR‐γ and CEBP‐α in the femoral head of rats in each group after treatment for 12 weeks. (d) Qualitative analyses of IHC. Data are presented as mean ± standard deviation. ^**^ *P* < 0.01 compared with the Control group and up‐LINC00473 + BMSCs + PLGA + SONFH group. CEBP‐α, CCAAT/enhancer‐binding protein‐α; HE staining, hematoxylin‐eosin staining; IHC, immunohistochemistry; LINC00473, LINCRNA‐00473; PLGA, polylactic‐co‐glycolic acid; PPAR‐γ, peroxisome proliferator‐activated receptor‐γ; rBMSCs, rat‐derived bone marrow mesenchymal stem cells; SONFH, Steroid‐induced osteonecrosis of the femoral head

Hematoxylin‐eosin (HE) staining was used to assess histological deformities. Rats in the SONFH and BMSCs + SONFH groups displayed osteonecrosis, characterized by: smaller subchondral trabeculae bone in the femoral head, sparser and pyknotic nuclei and empty lacunae, accompanied by the accumulation of adipocytes and fibrous tissues in the medullary cavity. These deformities were rarely found in the up‐LINC00473 + BMSCs + PLGA + SONFH group (Figure 11a). Besides, the rate of empty lacunae was calculated by counting in six fields randomly selected in one section. Our data suggested that the rate of empty lacunae in the up‐LINC00473 + BMSCs + PLGA + SONFH group was markedly lower than that of the SONFH and BMSCs + SONFH groups (Figure 11b).

Meanwhile, the expression of osteogenic markers (OPN3 and Runx‐2) and adipogenic markers (PPAR‐γ and CEBP‐α) in the femoral head of rats in each group was evaluated by immunohistochemistry (IHC) analysis. Our results revealed a higher expression of OPN3 and Runx‐2 coupled with a lower expression of PPAR‐γ and CEBP‐α in the up‐LINC00473 + BMSCs + PLGA + SONFH group, compared with those of the SONFH and BMSCs + SONFH groups (Figure 11c,d), suggesting that the osteogenesis was increased and adipogenesis was reduced in the femoral head of up‐LINC00473 + BMSCs + PLGA + SONFH rats.

Taken together, these findings provide evidence that the transplantation of PLGA hydrogels loaded with rBMSCs overexpressing LINC00473 could significantly promote bone repair and reconstruction in the necrotic area of the femoral head of SONFH rats. In addition, it should be noted that the above imaging and pathological abnormalities in the subchondral area of the femoral head were alleviated in the Vector + BMSCs + PLGA + SONFH and BMSCs + PLGA + SONFH groups when compared to those of the BMSCs + SONFH group. We speculate that this is due to a cell‐carrying effect of PLGA hydrogels. Therefore, the effects of the PLGA hydrogel on the migration of transplanted cells were evaluated.

### 2.11 Transplanted rBMSCs encapsulated within the PLGA hydrogel migrated from the medullary cavity to the femoral head of SONFH rats

**FIGURE 12:**
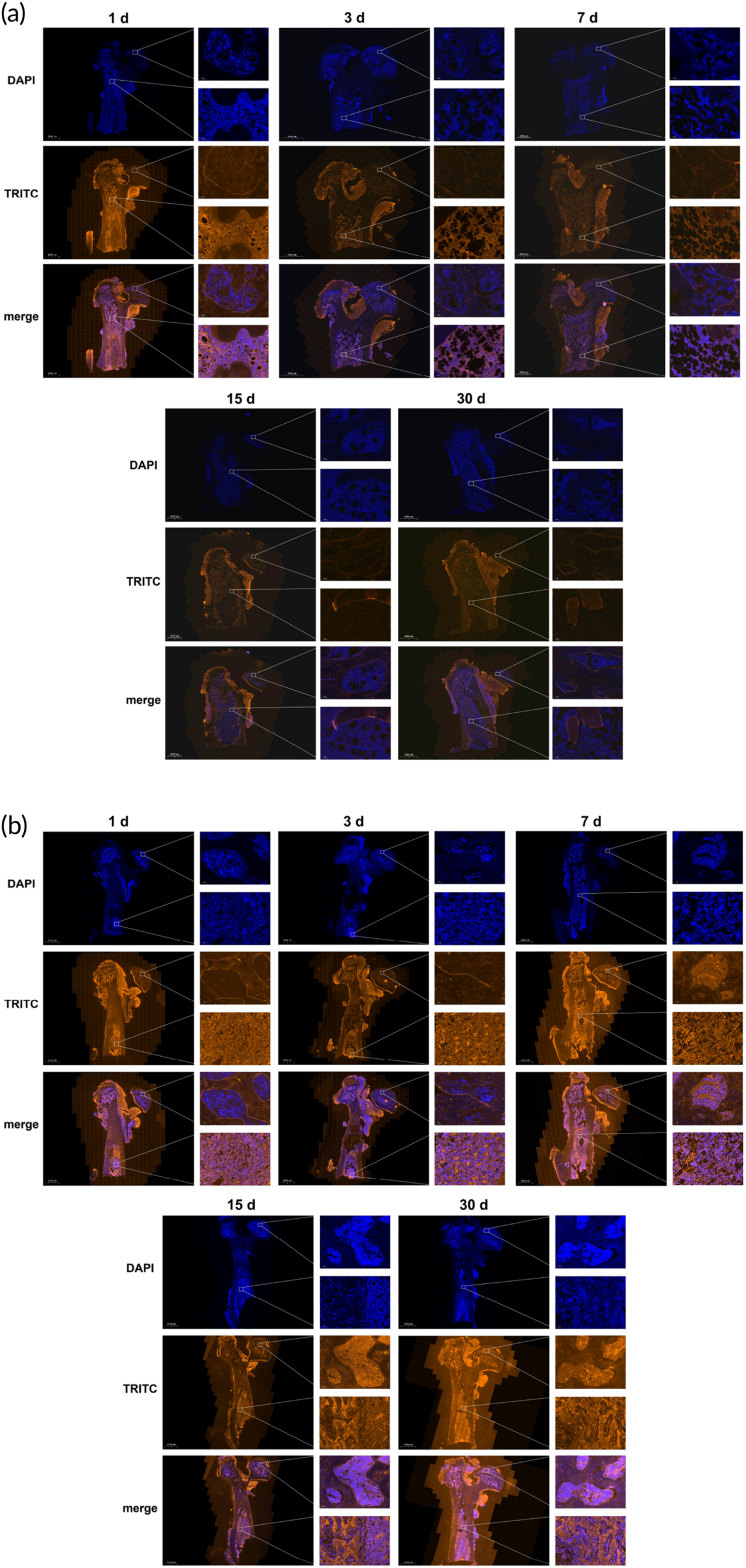
Tracking of transplanted rBMSCs in rats with SONFH. (a) Representative images showing the distribution of positive cells for luciferase in the femoral medullary cavity and femoral head of rats in the BMSCs + SONFH group after transplantation for 1, 3, 7, 15, 30 days, based on the IF staining. (b) Representative images showing the distribution of positive cells for luciferase in the femoral medullary cavity and femoral head of rats in the BMSCs + PLGA + SONFH group after transplantation for 1, 3, 7, 15, 30 days, based on the IF staining. IF staining, immunofluorescence staining; PLGA, polylactic‐co‐glycolic acid; rBMSCs, rat‐derived bone marrow mesenchymal stem cells; SONFH, Steroid‐induced osteonecrosis of the femoral head

To further investigate the effect of the PLGA hydrogel on the fate of transplanted rBMSCs, the cells were labeled by transfection with luciferase lentivirus in vitro, then cells were encapsulated or not in thermosensitive PLGA hydrogels, and were transplanted into the femoral medullary cavity of SONFH rats. After transplantation for 1, 3, 7, 15, or 30 days, immunofluorescence (IF) staining was used to evaluate the distribution of positive cells for luciferase. Our findings showed that in the BMSCs + SONFH group, the positive cells for luciferase were widely distributed in the medullary cavity after transplantation for 1 and 3 days, but the number of cells decreased gradually from the seventh day. Notably, positive cells for luciferase were not found in the femoral head up to 30 days after transplantation (Figure 12a). In the BMSCs + PLGA + SONFH group, positive cells for luciferase were only found in the medullary cavity after up to 3 days of transplantation. Surprisingly, positive cells began to adhere to the femoral bone marrow cavity wall, and were observed in the femoral head from the seventh day. Moreover, the number of positive cells in the femoral head and medullary cavity increased gradually from 7 to 30 days after transplantation (Figure 12b). The above results indicated that, compared with the transplantation of BMSCs alone, the transplanted rBMSCs encapsulated within thermosensitive PLGA hydrogels could migrate from the medullary cavity to the femoral head, which may be related to the PLGA hydrogel providing a suitable environment for harboring rBMSCs, thus avoiding apoptosis, necrosis, and absorption.

## 3 DISCUSSION

Based on our previous studies,^40^, ^41^ LINC00473 is a differentially expressed lncRNA in BMSCs from patients with SONFH and plays a key role in regulating the biological function of BMSCs. Here, we first investigated the effect of LINC00473 on the osteogenic and adipogenic differentiation of hBMSCs, and found that overexpression of LINC00473 significantly increased osteogenic differentiation, while it decreased adipogenic differentiation of hBMSCs. Genetical knockdown of LINC00473 reversed these effects, indicating that LINC00473 promoted osteogenesis and suppressed the adipogenesis of hBMSCs.

The role of the Wnt/β‐catenin signaling pathway in the osteogenesis and adipogenesis of BMSCs has been confirmed by an increasing number of studies.^54^, ^55^, ^56^, ^57^ The Wnt/β‐catenin signaling pathway is thought to be a signal cascade reaction between LRP5, β‐catenin, and TCF. Activation of LRP5 can increase the stable accumulation of β‐catenin in the cytoplasm and thereby up‐regulate the expression of TCF in the nucleus, which eventually increases the activation of the Wnt/β‐catenin signaling pathway.^57^ In the present study, overexpression of LINC00473 leads to a remarkable increase in the expression of LRP5, β‐catenin, and TCF1, which promoted osteogenesis and suppressed adipogenesis of hBMSCs. This effect was antagonized by DKK1 (a specific inhibitor of the Wnt/β‐catenin signaling pathway), reiterating that LINC00473 activated the Wnt/β‐catenin signaling pathway.

Notably, our previous works have demonstrated that miR‐23a‐3p was an upstream regulator of LRP5, and could inactivate the Wnt/β‐catenin signaling pathway to inhibit the osteogenic differentiation of hBMSCs.^38^ Therefore, we explored the role of miR‐23a‐3p in the LINC00473‐activated Wnt/β‐catenin signaling pathway. Our results suggested that the effects of LINC00473 were significantly weakened by miR‐23a‐3p mimics, indicating that miR‐23a‐3p mediated the activation of the Wnt/β‐catenin signaling pathway induced by LINC00473.

Increased apoptosis and decreased proliferation of BMSCs induced by long‐term exposure to glucocorticoids are considered another potential pathogenesis for SONFH.^13^, ^14^ In our previous studies, LINC00473 was shown to rescue hBMSCs from Dex‐induced apoptosis through activating the PEBP1/Akt/Bad/Bcl‐2 signaling pathway.^41^ In the present study, we observed that the overexpression of LINC00473 protected hBMSCs exposed to Dex through the increase of the expression of p‐Akt, p‐Bad, PEBP1 and Bcl‐2, which was in line with our previous reports.^41^ Remarkably, these effects of LINC00473 were suppressed by miR‐23a‐3p mimics. Moreover, the targeted binding between miR‐23a‐3p and PEBP1 was verified in the present study, suggesting that miR‐23a‐3p is involved in LINC00473‐induced activation of the PEBP1/Akt/Bad/Bcl‐2 signaling pathway in hBMSCs exposed to Dex.

The above results demonstrated that miR‐23a‐3p mediated the LINC00473‐activated Wnt/β‐catenin and PEBP1/Akt/Bad/Bcl‐2 signaling pathways to regulate the osteogenesis, adipogenesis and Dex‐induced apoptosis of hBMSCs. It should be noted that the functions of lncRNAs depend on their localization in cells. Indeed, lncRNAs located in the cytoplasm can affect the distribution of miRNA induce a post‐transcriptional regulation, acting as ceRNAs.^34^, ^35^, ^36^ Based on our previous FISH experiment, showing that LINC00473 was present in both cytoplasm and nucleus of hBMSCs,^40^ we further investigated the mutual regulation between LINC00473 and miR‐23a‐3p, and found that there was a negative interaction between LINC00473 and miR‐23a‐3p in hBMSCs. We proved that the specific sequence of miR‐23a‐3p could bind to the LINC00473 sequence. Interestingly, the Ago2 protein mediates the interaction between lncRNAs and miRNAs.^47^, ^48^, ^49^ In this study, RIP and RAP assays revealed that the interaction between LINC00473 and miR‐23a‐3p occurred in an AGO2‐dependent manner.

LINC00473 is reported to be a primate‐specific lncRNA and not normally expressed in rodents.^51^ In spite of this, the biological function of LINC00473 in mice should still be explored based on the homologous function induced by the overexpression of LINC00473.^50^ Therefore, we first investigated the functions of LINC00473 on the differentiation, migration, Dex‐induced apoptosis of rBMSCs, and found similar effects to those observed in hBMSCs.

Stem cell transplantation combined with tissue engineering has shown a promising prospect in the treatment of SONFH.^44^, ^53^ Injectable thermosensitive PLGA hydrogels are often used as cell‐delivery carriers in tissue engineering.^45^, ^46^ In this study, PLGA hydrogels showed reliable cell biocompatibility and could provide a suitable environment for harboring rBMSCs, based on the investigation of rBMSCs morphology in PLGA hydrogels and the assessment of toxicity of PLGA hydrogels on rBMSCs. Therefore, to further reveal the biological function of LINC00473 in vivo, we used PLGA hydrogels to encapsulate rBMSCs overexpressing LINC00473, and then transplanted these into a rat model of SONFH. Based on MRI, micro‐CT scanning and pathological analysis, we found that transplantation of PLGA hydrogels loaded with rBMSCs overexpressing LINC00473 promoted bone repair and reconstruction within the necrotic area of the femoral head of SONFH rats, compared with the effects of the transplantation of PLGA hydrogels loaded with control rBMSCs, that LINC00473 promoted osteogenesis and inhibited adipogenesis in vivo.

The fate of transplanted cells attracts considerable attention in cell therapy research. BMSCs could migrate into injured sites, and then differentiate into local components which substitute the damaged tissue.^58^ To our knowledge, several chemokines, cytokines, and growth factors, such as stromal derived factor‐1, osteopontin (OPN), basic fibroblast growth factor, vascular endothelial growth factor‐A, as well as signal pathways including the FAK, ERK, PI3K/Akt pathway were involved in cell migration of BMSCs.^58^ Our previous study has showed that up‐regulation of LINC00473 effectively relieved Dex‐induced migration inhibition of hBMSCs, confirming the beneficial effect of LINC00473 on the migration of hBMSCs.^38^ Similarly, our present study suggested that overexpression of LINC00473 could promote the migration of BMSCs to the scratch wound area in vitro. It was previously reported that LINC00473 suppressed migration and invasion of gastric cancer cells through regulating expression of Matrix metalloproteinase (MMP)‐2, MMP‐9, E‐cadherin, and Vimentin.^59^

Moreover, another key factor affecting the fate of transplanted cells was harboring effect of carrier. It was previously reported that over 90% of bone‐injected cells escaped from the bone marrow into the bloodstream and were found in the liver, lung, and spleen, although the injection of stem cells directly into the bone marrow was more effective to concentrate cells within the bone marrow compared with intravenous injections.^60^ In our study, we investigated the distribution of rBMSCs in the femur of rats with SONFH after intramedullary transplantation, and found that the transplanted rBMSCs encapsulated within the PLGA hydrogel could migrate from the medullary cavity to the necrotic area of the femoral head, an effect not found in the transplantation of rBMSCs alone. To further reveal whether PLGA hydrogel affects the migration of rBMSCs, we performed a scratch wound healing assay and found that PLGA hydrogels maceration extract had no positive effects on the migration of rBMSCs. Therefore, based on the three‐dimensional network structure and the reliable cell biocompatibility, we speculated that PLGA hydrogel could provide a suitable environment for harboring rBMSCs to avoid apoptosis, necrosis, and absorption, which may be beneficial to the homing of rBMSCs.

## 4 CONCLUSIONS

In summary, this study revealed underlying mechanisms of LINC00473 on the regulation of osteogenesis, adipogenesis, and Dex‐induced apoptosis of BMSCs. LINC00473 regulated two signaling pathway axes (miR‐23a‐3p/LRP5/Wnt/β‐catenin and miR‐23a‐3p/PEBP1/Akt/Bad/Bcl‐2). Furthermore, this study showed that the transplantation of PLGA hydrogels loaded with BMSCs overexpressing LINC00473 could significantly attenuate the progression of SONFH in vivo, which could provide a new strategy for the treatment of SONFH.

## 5 MATERIALS AND METHODS

### 5.1 Construction of the ceRNA interaction network of LINC00473

The data of the ceRNA interaction network of LINC00473 were obtained by the section of “ceRNA‐Network,” and the target genes of miR‐23a‐3p were predicted through the section of “miRNA‐Target” in the ENCORI website (http://starbase.sysu.edu.cn/index.php) as well as the predicted binding sites of miR‐23a‐3p on LINC00473. Briefly, “miRNA‐Target” was selected from the navigation bar, and “miRNA‐lncRNA” was then selected. Subsequently, “human” was selected in the section of “Genome,” and a search for “hsa‐miR‐23a‐3p” was made in the section of “microRNA” to complete the identification of lncRNAs matching with miR‐23a‐3p. Then, “LINC00473” was typed in the section of “Search” to obtained the binding sites between the miR‐23a‐3p and the LINC00473, and “Pan‐Cancer” was selected to perform the Pan‐Cancer analysis across 32 types of cancers. Finally, the network diagram was created using Cytoscape V3.7.2 software (NRNB, USA).

### 5.2 hBMSCs isolation and culture

The hBMSCs were isolated from the bone marrow tissue as previously reported.^40^ The third passage cells were utilized in subsequent experiments.

### 5.3 Phenotyping of hBMSCs

Flow cytometry was used to investigate the expression of hBMSCs surface markers (CD34, CD44, CD45, CD73, and CD90) using an Apogee A50‐MICRO flow cytometer (Apogee, UK) as previously reported.^40^

### 5.4 Cell transfection

Recombinant lentiviruses overexpressing LINC00473 (up‐LINC00473), short‐hairpin RNAs (shRNA) against LINC00473 (sh‐LINC00473), and negative control oligonucleotides (Vector and sh‐Control) were constructed by OBiO Technology (Shanghai, China) and transfected into hBMSCs as previously reported.^40^ The miR‐23a‐3p mimics and inhibitors were purchased from RiboBio Corporation (Guangzhou, China) and transfected into hBMSCs using Lipofectamine 2000 (Invitrogen, USA) as previously reported.^40^ The efficiency of transfection was determined by qRT‐PCR after 72 h. Three samples were analyzed in each group and three replicates were made per experiment.

### 5.5 Osteogenic and adipogenic differentiation of hBMSCs

Third passage hBMSCs were cultured in osteogenic and adipogenic differentiation medium (Fuyuanbio, Shanghai, China) as previously reported.^61^

### 5.6 ALP staining assay

The potential hBMSCs osteogenic differentiation was assessed using an ALP Staining kit (Solarbio, Beijing, China) as previously reported.^61^ Three samples were analyzed in each group and three replicates were made per experiment.

### 5.7 ALP activity assay

The ALP activity of hBMSCs was performed using the ALP Activity Assay kit (Nanjing Jiancheng Bioengineering Institute, Nanjing, China) as previously reported.^61^ Three samples were analyzed in each group and three replicates were made per experiment.

### 5.8 Oil Red O staining assay

The potential hBMSCs adipogenic differentiation was assessed using Oil Red O staining as previously reported.^61^ Three samples were analyzed in each group and three replicates were made per experiment.

### 5.9 TG content assay

Cellular TG content was detected using a Triglyceride Determination kit (Solarbio, Beijing, China) as previously reported.^61^ Three samples were analyzed in each group and three replicates were made per experiment.

### 5.10 Cell proliferation assay for hBMSCs

The proliferation of hBMSCs in the presence of Dex (10^−6^ mol/L) was evaluated by a Cell Counting Kit‐8 (CCK‐8 assay kit) (Solarbio, Beijing, China) as previously reported.^40^ Three samples were analyzed in each group and three replicates were made per experiment.

### 5.11 Assessment of the morphology of apoptotic cells in hBMSCs exposed to Dex

The morphology of apoptotic cells in the presence of Dex (10^−6^ mol/L) was assessed using the chromatin dye Hoechst 33342 kit as previously reported.^40^ Three samples were analyzed in each group and three replicates were made per experiment.

### 5.12 Flow cytometry measurement of apoptosis

Apoptotic cells were analyzed by flow cytometry using an Annexin V‐PE/7‐AAD kit as previously reported.^40^ Three samples were analyzed in each group and three replicates were made per experiment.

### 5.13 Total RNA isolation and qRT‐PCR analysis

Total RNA isolation, reverse transcription, and qRT‐PCR were conducted as previously reported.^40^ The relative expression of genes was evaluated by the 2^−△△Ct^ method. LINC00473 mRNA was normalized to GAPDH, while miR‐23a‐3p was normalized to U6. The sequences of primers are listed in Table 2. Three samples were analyzed in each group and three replicates were made per experiment.

**TABLE 2:** The sequences of primers for quantitative real‐time polymerase chain reaction

Gene	Primer sequences
BSPII	Forward: 5′‐CAGAGGAGGCAAGCGTCACT‐3′ Reverse: 5′‐CTGTCTGGGTGCCAACACTG‐3′
OPN3	Forward: 5′‐ACTCGAACGACTCTGATGATGT‐3′ Reverse: 5′‐GTCAGGTCTGCGAAACTTCTTA‐3′
Runx‐2	Forward: 5′‐TGTCATGGCGGGTAACGAT‐3′ Reverse: 5′‐AAGACGGTTATGGTCAAGGTGAA‐3′
PPAR‐γ	Forward: 5′‐CCTATTGACCCAGAAAGCGATT‐3′ Reverse: 5′‐CATTACGGAGAGATCCACGGA‐3′
CEBP‐α	Forward: 5′‐CTTCAGCCCGTACCTGGAG‐3′ Reverse: 5′‐GGAGAGGAAGTCGTGGTGC‐3′
FABP4	Forward: 5′‐TCCCTACTTGTGTGGCGTGAA‐3′ Reverse: 5′‐TCACCCGAGTGGTAGTCACAATG‐3′
LRP5	Forward: 5′‐TGGCCCGAAACCTCTACTG‐3′ Reverse: 5′‐GCACACTCGATTTTAGGGTTCT‐3′
β‐catenin	Forward: 5′‐CAATGGCTTGGAATGAGACT‐3′ Reverse: 5′‐CCCATCTCATGTTCCATCA‐3′
TCF1	Forward: 5′‐CCCACCAAGCAGGTCTTCAC‐3′ Reverse: 5′‐AAGGTCTCGATGACGCTGTG‐3′
GAPDH	Forward: 5′‐GGTCACCAGGGCTGCTTTTA‐3′ Reverse: 5′‐GGATCTCGCTCCTGGAAGATG‐3′
LINC00473	Forward 5′‐GGCAGCCTCAGGTTACAAAT‐3′ Reverse 5′‐AGGAGCAGGTAGGGAAATGA‐3′
U6	Forward: 5′‐CTCGCTTCGGCAGCACA‐3′ Reverse: 5′‐ACGCTTCACGAATTTGCGT‐3′

### 5.14 Western blotting analysis

Total protein extraction and western blotting analysis were performed as previously reported.^41^ The primary antibodies for OPN3, BSPII, Runx‐2, PPAR‐γ, CEBP‐α, FABP4, β‐catenin, LRP5, TCF, and AGO2 were purchased from Cell Signaling Technology (Danvers, USA) and diluted at an appropriate ratio according to the manufacturer's instructions. The primary antibody for GAPDH and all second antibodies were purchased from Elabscience (Shanghai, China).

### 5.15 Dual‐luciferase reporter assay system

All GV272‐LINC00473 vectors including GV272‐LINC00473‐WT and GV272‐LINC00473‐MUT, GV272‐PEBP1 vectors including GV272‐PEBP1‐WT and GV272‐PEBP1‐MUT, and the renilla luciferase plasmid were provided by Genechem company (Shanghai, China). The third passage cells were seeded in 96‐well plates at 2 × 10^4^ cells/well and transfected with GV272‐LINC00473 vectors/GV272‐PEBP1 vectors, renilla luciferase plasmid, miR‐23a‐3p mimics, and miR‐NC using Lipofectamine 2000. Forty‐eight hours after transfection, the expression of luciferase in each group was detected by the Dual‐luciferase assay kit (Yeasen biology, Shanghai, China) according to the manufacturer's instructions.

### 5.16 AGO2‐RIP assay

The RIP assay was performed using a RIP kit (BersinBio, Guangzhou, China) according to the manufacturer's instructions. Briefly, 4 × 10^7^ cells were lysed in polysome lysis buffer containing protease and RNase inhibitors, and were incubated with RIP buffer containing magnetic beads conjugated with anti‐AGO2 antibodies (Cell Signaling Technology, Danvers, USA) and immunoglobulin G (IgG) antibodies (BersinBio, Guangzhou, China) for 16 h at 4°C. Then, the proteinase K was used to digest the unbound proteins, and co‐immunoprecipitated RNA was isolated. Subsequently, the expression of LINC00473, miR‐23a‐3p, GAPDH, and U6 in each group were detected by qRT‐PCR.

### 5.17 RAP assay

RAP assay was performed using a RAP kit (Bersinbio, Guangzhou, China) according to the manufacturer's instructions. LINC00473 probes for the RAP assay were constructed by Bersinbio. Briefly, 4 × 10^7^ cells were cross‐linked with 1% formaldehyde and 0.4 g Glycine. Then, cell pellets were collected and lysed with lysis buffer containing protease and RNase inhibitors. After removing DNA, all samples were hybridized in 2 × hybridization buffer, and incubated with LINC00473 probes and streptavidin beads. After being eluted with protein elution buffer and DTT, the protein samples were used to detect the expression of AGO2 and GAPDH by western blotting. After being eluted and purified, the RNA samples were used to detect the expression of miR‐23a‐3p and U6 by qRT‐PCR.

### 5.18 rBMSCs isolation and culture

The rBMSCs were isolated from bone marrow in the bilateral femurs and tibias of Sprague‐Dawley rats as previously reported.^62^ The third passage cells were utilized in subsequent experiments.

### 5.19 Scratch wound assay

Cells at 5 × 10^4^ cells/well were seeded into 6‐well plates after rBMSCs were treated according to the groups, and then starved overnight. Cells were scraped with a tip after monolayer fusion and then incubated under conditions of 5% CO_2_ at 37°C. After cells were cultured for 0, 6, and 12 h, the wound widths were observed under an inverted phase contrast light microscope (Olympus CKX41, Japan), and the migration rate was calculated by the ImageJ software (version 1.52u). Three samples were analyzed in each group and three replicates were made per sample.

### 5.20 Morphological observation of rBMSCs in hydrogels

The PLGA hydrogel was dissolved in PBS at 1:5, and was filtered with a 0.22 μm membrane. To investigate the morphology of rBMSCs on the surface of PLGA hydrogels, 5 × 10^4^ cells/well were seeded on the surface of 1 ml PLGA hydrogel in 24‐well plates, and were observed under an inverted phase contrast light microscope (Olympus CKX41, Japan).

To investigate the morphology of rBMSCs within PLGA hydrogels, cells were encapsulated in PLGA hydrogels at 1 × 10^6^ cells/ml, and were cultured in DMEM containing 10% FBS under 5% CO_2_ at 37°C. Subsequently, the cells were fixed with 2.5% glutaraldehyde for 3 h and were observed using a SEM equipped with a temperature‐controlled sample holder.

### 5.21 Preparation of PLGA hydrogel maceration extract

The PLGA hydrogel was dissolved in PBS at 1:5, and was soaked in medium containing 10% FBS under 5% CO_2_ and 37°C. After being filtered with a 0.22 μm membrane, the PLGA hydrogel maceration extract was incubated with cells to assess cell proliferation and apoptosis.

### 5.22 Crystal violet staining assay

The third passage cells were seeded in 96‐well plates at 2 × 10^4^ cells/well and treated with the PLGA hydrogel maceration extract for 7 days. Cells cultured in medium containing 10% FBS were used as control. Then, cells were fixed with 4% paraformaldehyde for 10 min, and stained with 0.25% crystal violet solution for 30 min. Cells were observed under an inverted phase contrast microscope after washing three times. Subsequently, 200 μL methanol was added to each well to dissolve crystal violet, and the absorbance of each well was detected by a microplate reader (Tecan, Austria) at 570 nm. Three samples were analyzed in each group and three replicates were made.

### 5.23 Rats model of SONFH and grouping

All animal experiments were performed in a specific pathogen‐free grade animal laboratory, following the guidelines for the Care and Use of Laboratory Animals. A total of 80 healthy male Sprague‐Dawley (SD) rats aged 8‐week‐old with body weight of 300–350 g were provided by the Pengyue experimental animal breeding limited company (Jinan, China) and were used in this study. All rats had free access to conventional chow and tap water. A total of 60 rats were randomly divided into six groups. (1) SONFH group: Received intramuscular injection of 20 mg/kg/day MPS (Pfizer, New York, USA) 3 days/week for 3 weeks (*n* = 10); (2) up‐LINC00473 + BMSCs + PLGA + SONFH group: After 3 weeks of treatment according to the SONFH group, rats were treated with femoral intramedullary transplantation of PLGA hydrogels loaded with rBMSCs overexpressing LINC00473 at a final density of 1 × 10^6^ cell/ml (*n* = 10); (3) Vector + BMSCs + PLGA + SONFH group: After 3 weeks of treatment according to the SONFH group, rats were treated with femoral intramedullary transplantation of PLGA hydrogels loaded with rBMSCs transfected with vector at a final density of 1 × 10^6^ cell/ml (*n* = 10); (4) BMSCs + PLGA + SONFH group: after 3 weeks of treatment according to the SONFH group, rats were treated with femoral intramedullary transplantation of PLGA hydrogels loaded with rBMSCs at a final density of 1 × 10^6^ cell/ml (*n* = 10); (5) BMSCs + SONFH group: after 3 weeks of treatment according to the SONFH group, rats were treated with femoral intramedullary transplantation of rBMSCs suspended in PBS at a final density of 1 × 10^6^ cell/ml (n = 10); (6) Control group: Rats were treated with normal saline (n = 10). All surgeries were performed under anesthesia to minimize the suffering, and the appropriate volume of medullary cavity graft was 0.2 ml. After treatment for 12 weeks, the rats were euthanized by carbon dioxide asphyxiation, and the femoral heads were collected and examined by MRI, micro‐CT, HE staining, and IHC.

In addition, 20 rats were randomly divided into two groups (BMSCs + PLGA + SONFH group and BMSCs + SONFH group) and treated as described above. After treatment for 1, 3, 7, 15, 30 days, the rats were euthanized by carbon dioxide asphyxiation, and the femoral heads in each group were collected and examined by immunofluorescence.

### 5.24 MRI analysis

MRI with a phased‐array body coil (Skyra 3.0T MRI System, Siemens, Germany) was performed to determine abnormal signals of the femoral head in each group. The turbo spin‐echo T_2_‐weighted fat‐saturated images in the transverse plane were obtained with the following parameters: repetition time (3000 ms), echo time (38 ms), slice thickness (1.4 mm), interslice distance (0.14 mm), field of view (120 mm), and matrix (384 × 320 pixels).

### 5.25 Micro‐CT analysis

The femoral heads fixed with 4% paraformaldehyde were analyzed by μCT100 (SCANCO MEDICAL, Zurich, Switzerland). Micro‐CT scanning was set according to the following parameters: scanning energy intensity (70 KVp, 200 μA), filter (0.5 AL), matrix (1022 mm × 1022 mm), field of view (15.1 mm), resolution (14.8 μm), and sampletim (250 ms). Two‐dimensional images were obtained automatically using the micro‐CT system. The analysis data for trabecular bone in the femoral head, including Tb.Th, Tb.Sp, BV/TV, and Tb.N, as well as the three‐dimensional images were obtained by SCANCO μCT Evaluation Program V6.6 software.

### 5.26 Histological analysis

The femoral heads in each group were fixed in 4% paraformaldehyde and decalcified by 10% ethylenediaminetetraacetic acid. After decalcification for 4 weeks, the specimens were dehydrated and embedded in paraffin. Subsequently, 5 μm paraffin sections in the coronal plane were made by a microtome (Leica, Biocut, German), and slices stained with hematoxylin and eosin to investigate the subchondral structure. In addition, several sections were deparaffinized and used for the immunohistochemistry analysis of the expression of OPN3, Runx2, PPAR‐γ, and CEBP‐α. Furthermore, sections obtained from the rats treated for 1, 3, 7, 15, 30 days in BMSCs + PLGA + SONFH group and BMSCs + SONFH group, were used in the immunofluorescence analysis for the expression of luciferase. Photomicrographs were obtained by a panorama scanner (3DHISTECH P250 FLASH, Hungary).

### 5.27 Statistical analysis

The statistical analysis in this study was conducted by the SPSS 19.0 software (IBM, USA). The one‐way analysis of variances was preformed to compare the data of more than two groups. Values are expressed as mean ± standard deviation (SD). The homogeneity test for variance was performed. Tamhane's T2 test was adopted when heteroscedasticity was found. *P*‐values <0.05 were considered significant. The statistics charts were made by GraphPad Prism 8 software (GraphPad, CA, USA).

## CONFLICT OF INTEREST

The authors declare that they have no conflict of interest.

## AUTHOR CONTRIBUTIONS

**Yingxing Xu:** Conceptualization (equal); data curation (equal); formal analysis (equal); investigation (equal); methodology (equal); project administration (equal); software (equal); validation (equal); visualization (equal); writing – original draft (equal). **Yaping Jiang:** Data curation (equal); formal analysis (equal); investigation (equal); methodology (equal); software (equal); visualization (equal). **Yingzhen Wang:** Conceptualization (equal); methodology (equal); project administration (equal); supervision (equal). **Bin Jia:** Investigation (equal); methodology (equal); software (equal); validation (equal); visualization (equal). **Song Gao:** Investigation (equal); software (equal); validation (equal); visualization (equal). **Haiyang Yu:** Data curation (equal); formal analysis (equal); investigation (equal); methodology (equal). **Haining Zhang:** Methodology (equal); project administration (equal); supervision (equal). **Chengyu Lv:** Methodology (equal); project administration (equal); supervision (equal). **Haiyan Li:** Methodology (equal); software (equal); validation (equal); visualization (equal). **Tao Li:** Conceptualization (equal); funding acquisition (supporting); methodology (equal); project administration (equal); resources (equal); supervision (equal); writing – review and editing (equal).

## Data Availability

The data that support the findings of this study are available from the corresponding author upon reasonable request.
